# Surface analysis of cannabigerol cocrystals: linking crystal structure to enhanced properties

**DOI:** 10.1107/S2052252525001009

**Published:** 2025-02-27

**Authors:** Eliška Zmeškalová, František Stara, Tereza Havlůjová, Miroslav Šoóš

**Affiliations:** a Department of Chemical Engineering University of Chemistry and Technology Prague Technická 3 166 28Prague 6 Czechia; b Department of Structure Analysis Institute of Physics of the Czech Academy of Sciences Cukrovarnická 112/10 162 00Praha 6 Czechia; Formby, Liverpool, United Kingdom

**Keywords:** surfaces, particles, crystal structures, topology, cannabigerol, dissolution, crystal engineering, Cambridge Structural Database, *CSD-Particle*, cocrystals, properties of solids, crystal design

## Abstract

Cannabigerol is a bioactive compound derived from *Cannabis sativa*. It displays many promising pharmaceutical and nutraceutical properties. Its use and research are complicated by its thermally unstable solid form with low solubility and needle habit, preventing easy formulation into tablets or capsules. To overcome these problems, we conducted a crystallization screening with the aim to discover new crystal forms with enhanced properties. Though polymorph and solvate screenings did not yield new forms, the cocrystal screening was successful. Two cocrystals were discovered, one with piperazine and another with tetra­methyl­pirazine, both in a 1:1 ratio. The latter can exist in three polymorphic forms. Both offer improvements in the melting point and crystal habit, and the cocrystal with tetra­methyl­pirazine also shows a significant enhancement in dissolution rate. The new solid forms were analysed by a combination of methods, including X-ray powder diffraction, nuclear magnetic resonance spectroscopy, differential scanning calorimetry, thermogravimetric analysis and intrinsic dissolution rate. Single-crystal X-ray diffraction data were used to solve the crystal structures, which were then compared with that of pure CBG. The crystal morphologies and surfaces were comprehensively analysed using the *CSD-Particle* suite, with various properties correlated against dissolution rates. While surface attachment energy and roughness (rugosity) did not show significant effects, the concentration of unsatisfied hydrogen-bond donors displayed a positive correlation. There were two parameters with a very strong correlation to dissolution rate: the propensity for interactions with water molecules, determined by the maximum range in the full interaction maps on the surface calculated for the water probe, and also the difference in the positive and negative electrostatic charges. These parameters proved highly predictive of aqueous dissolution, offering immense utility in pharmaceutical development.

## Abstract 2

This study investigates the solid forms of cannabigerol and identifies two new cocrystals, showing significant improvements in physicochemical properties. The findings highlight the strong predictive capability of surface interaction parameters for aqueous dissolution, offering valuable insights for pharmaceutical development.

## 1. Introduction

Natural drugs represent an alternative to their chemically synthetized counterparts. Cannabinoids are a class of compounds found naturally in plants of the genus *Cannabis*. The medicinal effects of the *Cannabis* plant and its extract have been known to humans for millennia and, as such, were part of many religious and healing customs (Bonini *et al.*, 2018 ▸). The isolation of the individual compounds from cannabis extract in the 1960s and the subsequent discovery of the endocannabinoid system in the 1990s are the two main developments leading to the contemporary interest in cannabinoids as active pharmaceutical ingredients (APIs) (Bonini *et al.*, 2018 ▸). One of the promising pharmaceutical cannabinoids is cannabigerol (CBG). This non-psychoactive cannabinoid has been investigated for the treatment of several conditions and interest in this compound has been steadily growing since the 2000s (Anokwuru *et al.*, 2022 ▸).

**Figure 1:**
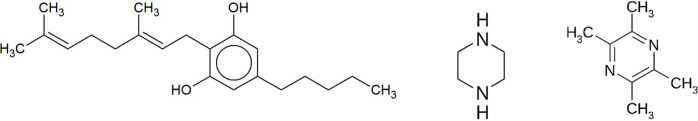
The structures of cannabigerol, piperazine and tetra­methyl­pirazine.

CBG is a non-psychotropic member of the cannabinoid family mainly found in plants of the genus *Cannabis*. CBG is biosynthesized by de­carboxyl­ation of cannabigerolic acid which serves as a common precursor to all cannabinoids (Tahir *et al.*, 2021 ▸). Together with other cannabinoids such as cannabidiol or tetra­hydro­cannabinol, it can interact with the endocannabinoid system in the human body through the cannabinoid receptors CB_1_ and CB_2_ (Navarro *et al.*, 2018 ▸). Several studies focused on the pharmacological effects of CBG suggest that it could be potentially used for its anti-inflammatory, anticancer, antibacterial, neuroprotective and appetite-stimulating properties (Deiana, 2017 ▸; Anokwuru *et al.*, 2022 ▸; Nachnani *et al.*, 2021 ▸). CBG has the molecular formula C_21_H_32_O_2_ and the molecular weight 316.5 g mol^−1^ (https://pubchem.ncbi.nlm.nih.gov/compound/5315659). Its sys­tematic name is 2-[(2*E*)-3,7-di­methyl­octa-2,6-dienyl]-5-pentyl­benzene-1,3-diol. The structure of CBG is presented in Fig. 1 ▸.

Safety and performance are the two main concerns during the development of any drug product from an API (Hilfiker, 2006 ▸). For drugs administered in solid form, a crucial step in this development is the solid form investigation (Brittain, 2009 ▸). This includes establishing whether the API can exist in multiple forms, and whether it is possible to improve the properties of the API by creating multicomponent solid forms (Storey & Ymén, 2011 ▸). Polymorphs are different crystalline forms of the same pure substance and can exhibit distinct physical properties such as melting point, solubility and stability (Bernstein, 2011 ▸; Skořepová *et al.*, 2013 ▸; Holaň *et al.*, 2016 ▸; Chatziadi *et al.*, 2020 ▸). These variations can significantly impact the bioavailability and manufacturability of a drug (Khadka *et al.*, 2014 ▸; Zvoníček *et al.*, 2018 ▸). Solvates are crystalline structures that incorporate solvent molecules into their lattice, potentially altering the solubility and stability of the API (Sládková *et al.*, 2015 ▸; Tieger *et al.*, 2016*b* ▸; Zvoníček *et al.*, 2017 ▸; Byrn *et al.* 2017 ▸). Hydrates, a specific type of solvates, include water molecules within their crystal structure, which can influence the hygroscopicity and dissolution rate of a drug (Braun & Griesser, 2016 ▸; Tieger *et al.*, 2016*a* ▸; Byrn *et al.*, 2017 ▸). Salts are formed by the reaction of an API with an appropriate counterion, often enhancing solubility and stability (International Union of Pure and Applied Chemistry, 2011 ▸; Skořepová *et al.*, 2016 ▸, 2017 ▸). Cocrystals, on the other hand, are crystalline materials composed of the API and one or more coformers, which are typically non-volatile compounds (Desiraju, 2003 ▸; Aitipamula *et al.*, 2012 ▸; Skořepová *et al.*, 2014 ▸; Sládková *et al.*, 2014 ▸, 2017 ▸). These coformers interact with the API through non-covalent bonds, leading to new solid forms with potentially improved physicochemical properties (Cheney *et al.*, 2011 ▸; Abramov *et al.*, 2012 ▸). The discovery and characterization of these solid forms are essential for optimizing the performance and manufacturability of pharmaceutical compounds (International Union of Pure and Applied Chemistry, 2011 ▸).

Crystallization screening is a systematic approach used to identify and characterize new solid forms of an API (Hilfiker, 2006 ▸; Anderton, 2007 ▸). This process involves varying the crystallization conditions, such as solvent, temperature and concentration, to explore the solid form landscape of the compound. In the case of CBG, its thermally unstable solid form (*T*_m_ 54°C; https://pubchem.ncbi.nlm.nih.gov/compound/5315659) with low solubility poses significant challenges for formulation into tablets or capsules (McKellar *et al.*, 2014 ▸). To address these issues, a comprehensive crystallization screening was conducted. The solid form investigation of CBG presented here expands on available solid-state data. There is currently one CBG crystal structure in the Cambridge Structural Database (Groom *et al.*, 2016 ▸) – pure CBG [ref. code UHIHEB (private communication)]. In this article, we denote this form as CBG I. As for multicomponent forms, there are mentions of CBG forming cocrystals with proline (Holland & Eberlin, 2021 ▸), betaine and carnitine (Tesson *et al.*, 2020 ▸) in the patent literature. However, no peer-reviewed data are available.

Our aim in this study was to prepare solid forms of CBG with improved properties and to understand how these bulk properties stem from the crystal structure (Pallikara *et al.*, 2024 ▸). Recently, the Cambridge Crystallographic Data Centre has released the *CSD-Particle* (Moldovan & Maloney, 2024 ▸) module in the *Mercury* (Macrae *et al.*, 2020 ▸) software, a powerful toolset designed to facilitate rapid assessment of crystalline particles’ mechanical and chemical properties using visual and statistical tools. *CSD-Particle* predicts particle shape and surface facets, providing insights into parameters such as hydrogen-bond (HB) donors and acceptors, surface chemistry, charge distributions, slip planes, and full interaction maps (FIMs) (Kopczyńska *et al.*, 2024 ▸). These parameters help us to understand particle wettability, stickiness, tabletability and flow characteristics (Prandini *et al.*, 2024 ▸). The tool visualizes surface chemistry and charge distributions, aiding in identifying HB donors and acceptors critical for determining wettability and electrostatic properties (Bryant *et al.*, 2019 ▸). By evaluating particle surface interactions, *CSD-Particle* offers insights into the mechanical properties of the particles, including rugosity, surface area and HB density. *CSD-Particle* also calculates and visualizes full interaction maps on the surface (FIMoS), which utilize interaction data from the CSD to search for surface interactions based on specific functional groups. These maps predict where interactions are most likely to occur on the crystal surface by indicating the probability of interaction between a molecular fragment and a given probe. By evaluating these interactions across the calculated surface, FIMoS provides detailed insights into surface chemistry. This includes assessments of hydro­philicity with water oxygen probes or hydro­phobicity with methyl carbon probes, providing a comprehensive understanding of surface interactions. Higher grid densities in FIMs suggest a greater likelihood of finding particular interactions beyond random chance. For example, a range value of 75 means that, in that region, the density of contacts in the underlying CSD data is 75 times more than random. Additionally, the tool facilitates the assessment of internal crystal lattice interactions and hydrogen-bonding dimensionality, offering insights into how internal bonding affects surface properties and mechanical behaviour.

## 2. Experimental

### 2.1. Chemicals

CBG used for this study was purchased from Pharmabinoid in crystalline form and was stored in a fridge at 2–5°C. In total, 14 solvents and 2 solvent mixtures were used during the screening, all were used at room temperature. The solvents used, their distributors and properties are provided in Table S1 and the details of the solvent mixtures are shown in Table S2 in the supporting information. All coformers were purchased from Sigma–Aldrich. A list of these coformers is presented in Table 1 ▸.

### 2.2. Slow evaporation

50 mg of CBG was added to a 2 ml vial. In cocrystal screening experiments, the vial also included an equimolar amount of the coformer. The chosen solvent was added until all material dissolved while stirring on a magnetic stirring plate. After removing the magnetic stirrer, the vial was left inside a fume hood with the cap slightly ajar until all the solvent evaporated. The solid was transferred from the vial into a clean container and analysed using X-ray powder diffraction (XRPD).

### 2.3. Slurry mixing

50 mg of CBG was added to a 2 ml vial. In cocrystal screening experiments the vial also included equimolar amount of coformer. The chosen solvent was added so that the material was not dissolved and formed a suspension instead. The vial was closed tightly and sealed with laboratory film, and was inserted into an Eppendorf ThermoMixer C and mixed at 600 r.p.m. for seven days at room temperature. After seven days the solid phase was filtered and dried using a vacuum flask and a fritted glass funnel with a pore size of 3. The solid was transferred from the funnel into a clean container and analysed using XRPD.

### 2.4. Liquid-assisted grinding

Approximately 20 mg of CBG and an equimolar amount of coformer were added to a 2 ml polypropyl­ene vial. Two 5 mm stainless steel balls and 5 µl of cyclo­hexane were added to the vial. The vial was tightly closed and affixed in a Retsch MM400 mixer mill. The duration of milling was 20 min and the frequency was 20 Hz. The material without the milling balls was transferred into a clean container and analysed using XRPD.

### 2.5. X-ray powder diffraction

XRPD was conducted using the X’PERT3 POWDER PANalytical diffractometer. A copper *K*α emission (λ = 1.542 Å) X-ray source was used, with an acceleration voltage of 40 kV and an anode current of 30 mA. The range of measurement was 5–50° 2θ with a step size of 0.039° and a step time of 0.7 s. The thickness and area of the powder samples were 0.3 mm and 15 mm × 20 mm, respectively. The sample holder was a low-background silicon wafer. Primary ray correction was done using a 0.04 rad Soller slit, a 15 mm mask and an automatic divergence slit. Secondary ray correction was done using a 0.04 rad Soller slit and a 5.0 mm anti-scatter slit. An ultrafast 1D detector PIXcel PANalytical with 255 active channels was used.

### 2.6. Single-crystal X-ray diffraction

Crystals of a suitable size (around 0.2 mm) of the CBG I cocrystal were obtained by dissolving 20 mg of CBG in 200 µl of acetone with stirring and heating at 35°C and then letting the acetone slowly evaporate through a needle in the closed cap. Crystals of suitable size of the CBG–PIP (piperazine) cocrystal were obtained by dissolving 20 mg of CBG and an equimolar amount of PIP in 500 µl of ethyl acetate with stirring and heating at 35°C and then letting the ethyl acetate slowly evaporate through a needle in the closed cap. Suitable sized crystals of the CBG–TMP (tetra­methyl­pirazine) cocrystal form I were obtained by dissolving 50 mg of CBG and an equimolar amount of TMP in 400 µl of acetone and letting the acetone slowly evaporate from a slightly opened vial.

Single-crystal X-ray diffraction (SCXRD) measurements were performed at 95 K using a four-circle Rigaku Oxford Diffraction SuperNova diffractometer with a micro-focus sealed tube, mirror-collimated Cu *K*α radiation (λ = 1.54184 Å) and an Atlas S2 CCD detector. The data reduction and absorption correction were carried out using the *CrysAlis­Pro* software. The structures were solved by charge-flipping methods using the *Superflip* (Palatinus & Chapuis, 2007 ▸) software and refined by full-matrix least-squares on *F*^2^ using the *Crystals* (Betteridge *et al.*, 2003 ▸) and *Jana2020 ▸* (Petříček, 2023 ▸) software. The *MCE* software was used for the visualization of residual electron density maps (Rohlíček & Hušák, 2007 ▸). The hydrogen atoms were all located in a difference map, but those attached to carbon atoms were repositioned geometrically. The hydrogen atoms were initially refined with soft restraints on the bond lengths and angles to regularize their geometry (C—H in the range 0.93–0.98, N—H in the range 0.86–0.89 and O—H of 0.82 Å) and *U*_iso_(H) (in the range 1.2–1.5 × *U*_eq_ of the parent atom), after which the positions were refined with riding constraints (Betteridge *et al.*, 2003 ▸).

In the structure of CBG–PIP, there is disorder in one of the aliphatic chains of CBG. The occupancies were refined to 0.55:0.45. To achieve a reasonable model, the geometry of the fragments and the shapes of the ADPs were restrained to be similar.

The crystal structures were compared with respect to their CBG conformations and molecular packing in the software *CrystalCMP* (Rohlíček *et al.*, 2016 ▸; Rohlíček & Skořepová, 2020 ▸). It compares the structures and creates similarity dendrograms (Figs. S7 and S8). *CSD-Particle* (Moldovan & Maloney, 2024 ▸) lattice energies were determined using the attachment energy method as implemented in *VisualHabit* (Clydesdale *et al.*, 1991 ▸). The calculations employed the Dreiding II force field with Gasteiger charges and a limiting radius of 30 Å. The surface chemistry and topology of the calculated facet morphology were examined using *Surface Analysis* (Bryant *et al.*, 2019 ▸). Additionally, particle shapes were classified according to the Zingg methodology (Angelidakis *et al.*, 2022 ▸; Zingg, 1935 ▸).

### 2.7. Differential scanning calorimetry

Differential scanning calorimetry (DSC) was conducted using the Setaram DSC 131. The initial weight of samples was between 1 and 4 mg. The heating program consisted of 10 min at 25°C, followed by heating to 305°C with a constant heating rate of 5°C min^−1^. Measurements were carried out in air.

### 2.8. Thermogravimetric analysis

Thermogravimetric analysis (TGA) was conducted on a Stanton Redcroft TG-750 thermobalance. The initial weights of the samples were between 1 and 10 mg. The heating program started at 30°C and finished at 300°C with a constant heating rate of 5°C min^−1^. Measurements were carried out in air.

### 2.9. Nuclear magnetic resonance

Structural analysis was carried out using ^1^H nuclear magnetic resonance spectroscopy. Measurements were performed at 600 MHz using the Bruker 600 Avance III spectrometer or at 500 MHz using the Bruker Avance III 500 MHz spectrometer. The samples were dissolved in deuterated methanol, and the solvent also served as a chemical shift reference. Measurements were carried out at 298 K. The results from spectroscopy were evaluated using *TopSpin* (version 4.1.3) from Bruker.

### 2.10. Intrinsic dissolution rate

The intrinsic dissolution rate (IDR) was determined using a Sirius InForm device (Pion Inc. USA). IDR discs of 3 mm diameter were prepared by compressing powder material at a constant load of 60 kg for 1 min. The selected dissolution medium consisted of diluted hydro­chloric acid (pH 2) with the addition of 0.5% (*w*/*w*) of Tween 20, the volume used for each experiment was 40 ml. UV spectra were collected every 30 s using a probe with a 20 mm optical path length. Absorbance at 276 nm wavelength was used to evaluate the amount of API released at each time point; in the case of cocrystals with TMP, a correction had to be established by calculating the ratio between absorbance at 276 and 300 nm as both CBG and TMP absorb at the selected wavelength (267 nm). The IDR value was obtained from a linear fit of the experimental data with the exclusion of the first 10 data points, as the beginning of the experiment usually represents the dissolution of free powder stuck to the discs during the preparation process. Measurements were done in triplicates.

## 3. Results

### 3.1. Polymorph and solvate screening

For the polymorph and solvate screening, 16 samples were prepared using the slow evaporation method, and one sample was prepared using liquid-assisted grinding. The experiments did not produce new solid forms – all the samples exhibited crystallinity and remained in the starting solid form CBG I. Further details can be found in the supporting information.

### 3.2. Cocrystal screening

The cocrystal screening focused on testing 22 potential cocrystallization partners for CBG under a variety of crystallization conditions. The coformers for the screening were systematically selected to provide a good variety of molecules with a high chance of interaction with CBG. All three known CBG cocrystals in patents (with l-proline, betaine and carnitine) are formed with zwitterionic compounds, so they were one of our focuses. Arginine, valine, lysine, glutamic acid and glutamine were selected, for example. We also looked at other cannabinoids and their cocrystals. Tetra­methyl­pyrazine was selected because it is known to form a cocrystal with cannabidiol (Bernstein, 2011 ▸). We were further inspired by the CBG cocrystal with l-proline and selected coformers that had a somewhat similar molecular structure (cyclic/aromatic compounds with nitro­gen in the ring, optionally with a carb­oxy­lic group). Quercetin, polydatin, indole and piperazine were selected, for example. Our last path of the cocrystal design was through the examination of possible synthons (Skořepová *et al.*, 2013 ▸; Chatziadi *et al.*, 2020 ▸; Holaň *et al.*, 2016 ▸). CBG has two hydroxyl groups and one of the strongest heterosynthons for cocrystal preparation of alcohols is the alcohol–pyridine/pyridine-*N*-oxide synthon. Pyridine-*N*-oxide, 4-methyl­pyridin-*N*-oxide, isonicotinic acid *N*-oxide, isonicotin­amide and nicotinamide were selected. Other aromatic amides were also selected to test the alcohol–amide synthon (hippuric acid in addition to the above-mentioned amidic compounds). All selection criteria for each coformer are listed in Table 1 ▸.

**Figure 2:**
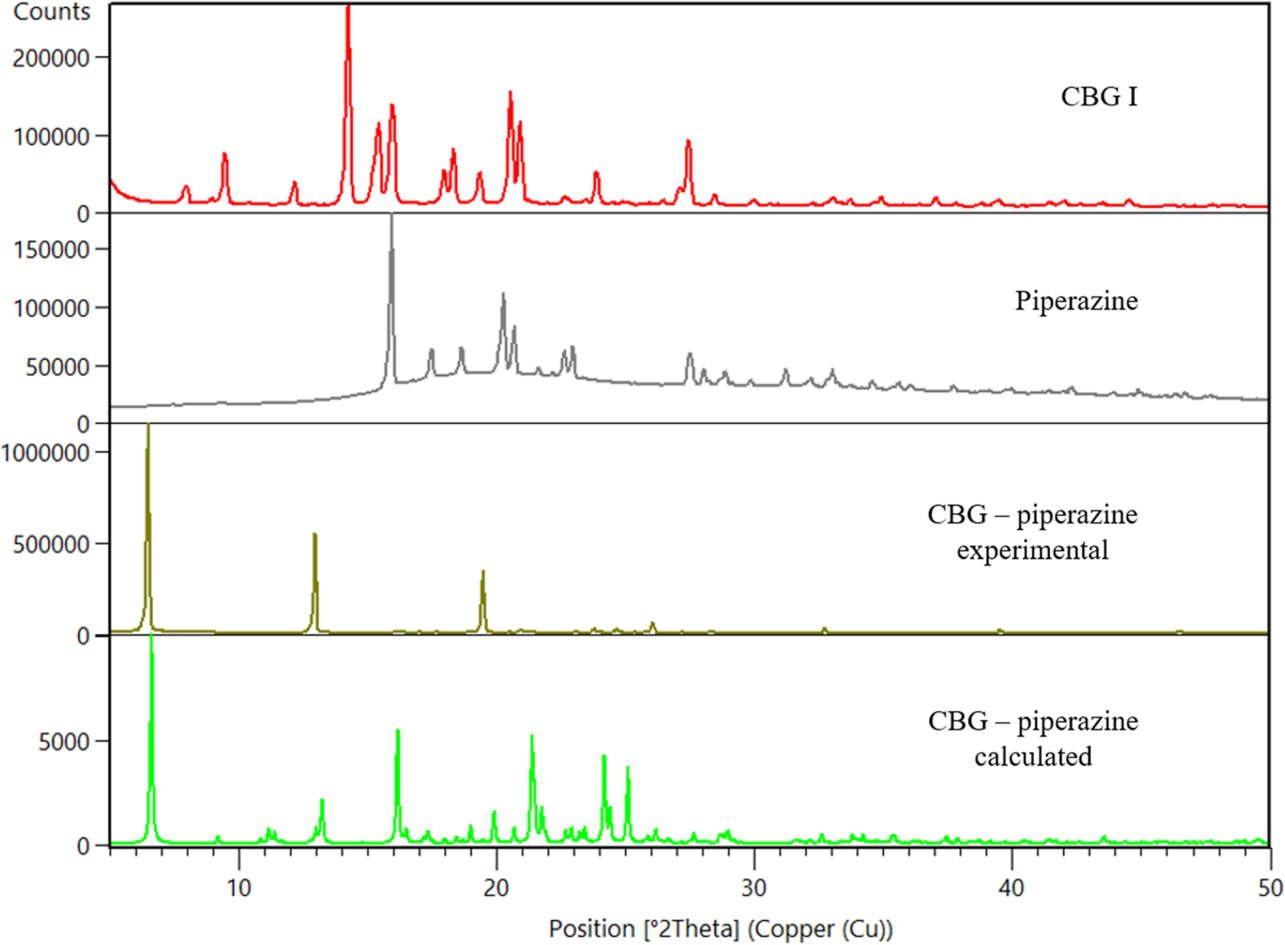
Comparison of powder diffractograms of CBG, PIP and a novel CBG–PIP solid phase.

Cocrystals with two coformers were discovered, with piperazine (PIP) and tetramethylpyrazine (TMP). The cocrystal with TMP exists in three polymorphic forms, I, II and III. CBG–TMP I seems to be the thermodynamically preferred form. Figs. 2 ▸–3 ▸ show the comparison between XRPD patterns of starting components, the cocrystals and their calculated patterns from the crystal structure. All details regarding the cocrystal screening experiments can be found in the supporting information.

#### 3.2.1. NMR spectroscopy

To further analyse the novel solid phases solution, ^1^H NMR spectra of the samples were measured to establish the stoichiometric ratios of CBG and coformers. Figs. S3–S6 display the spectra. Fig. S3 is the ^1^H NMR spectrum of the CBG–PIP solid phase. The intensities of the hydrogen signals reveal that the CBG to PIP ratio is 1:1, suggesting the formation of a cocrystal. Figs. S4–S6 show the ^1^H NMR spectra of the CBG and TMP solid phases. All three show the same composition, and the intensities of the hydrogen signals reveal that the ratio of CBG to TMP is 1:1 in all samples, suggesting the formation of a cocrystal in three polymorphic forms.

#### 3.2.2. Thermal analysis

The thermal properties of the new cocrystal samples were analysed using DSC and TGA. To investigate the change of thermal properties, the untreated CBG was analysed using DSC and TGA as well. The results of these measurements can be seen in Fig. 4 ▸.

All DSC curves contain a sharp peak representing melting. The CBG–PIP cocrystal melts at 126°C and the CBG–TMP cocrystal melts at 75°C in form I and at 76°C in forms II and III. In contrast, pure CBG melts at 54°C. The higher melting points of cocrystals suggest that their thermodynamic stability is higher than that of untreated CBG. The similar melting points of the three polymorphic forms of the CBG–TMP cocrystal suggest that their stability could be similar to each other. This may explain why, so far, we were not able to establish perfectly reliable preparation procedures for each of them.

All four TGA curves continue straight during initial heating, suggesting that there is no water or other solvent present in the samples. CBG decomposes from around 200°C to around 290°C, PIP decomposes from around 100°C to around 180°C and TMP decomposes from around 90°C to around 190°C, corresponding to the observed mass losses.

#### 3.2.3. Intrinsic dissolution

In many ways, the dissolution properties are the most crucial aspect of new pharmaceutical solid forms, having a great impact on bioavailability (Amidon *et al.*, 1995 ▸). As our work focuses on a compound of great interest to the pharmaceutical (Deiana, 2017 ▸; Jastrząb *et al.*, 2022 ▸) and nutraceutical (Kanabus *et al.*, 2021 ▸; Peters *et al.*, 2023 ▸) industries, we conducted intrinsic dissolution rate (IDR) measurements to assess the therapeutic potential of our cocrystals. For IDR, the powder under study is compressed into a disc with a defined surface area. Therefore, this analysis provides insight into the dissolution rate of the investigated solid form, while eliminating any potential influence from varying particle sizes. The IDR values of pure CBG as well as newly prepared cocrystals were established. The IDR of pure CBG was measured to be 16.92 ± 0.74 µg cm^−2^ min^−1^, a very similar result was also achieved for the PIP cocrystal as the value of 17.52 ± 1.19 µg cm^−2^ min^−1^ was calculated. On the other hand, the CBG–TMP I cocrystal measurement displays a significant increase in the dissolution rate with the value of 46.63 ± 2.47 µg cm^−2^ min^−1^, which is 2.9× higher than pure CBG (Figs. 5 ▸ and S9).

#### 3.2.4. Pharmaceutical acceptability

Piperazine and tetra­methyl­pyrazine are both promising cocrystal formers with CBG offering a combination of low toxicity, and established pharmaceutical use and acceptability. Table 2 ▸ summarizes some of the relevant information.

Piperazine is approved for use in various formulations and has a well established safety record. It is commonly used in pharmaceutical formulations as an anthelmintic agent (Vardanyan & Hruby, 2006 ▸). The therapeutic dose is approximately 30 mg kg^−1^ body weight per day. Piperazine has a low toxicity profile but can still pose some risks as a mild hepatotoxin and neurotoxin, with NOAEL (no observed adverse effect level) values identified as 25 mg kg^−1^ body weight per day for liver toxicity and 50 mg kg^−1^ body weight per day for neurotoxic effects (EU Risk Assessment Report, https://echa.europa.eu/documents/10162/35f9602c-cb84-448f-9383-250e1a5ad350).

Tetra­methyl­pirazine, used in traditional Chinese medicine (Chen *et al.*, 2017 ▸), exhibits low toxicity and has been studied for its antitumor and neuroprotective properties, with many studies showing its ability to reduce the toxicity of chemotherapy and to mitigate its side effects (Xu *et al.*, 2022 ▸). It is utilized in treatments for neurodegenerative diseases and cancer, with its antioxidative and anti-inflammatory properties enhancing its therapeutic potential. Its reported NOAEL is 55 mg kg^−1^ per day (Adams *et al.*, 2002 ▸).

When combined with CBG, if we assume a daily dose of 62.5 mg (https://healercbd.com/cbg-dosage-how-much-cbg-should-i-take/), the dose of PIP would be 0.18 mg kg^−1^ per day, and that of TMP 0.28 mg kg^−1^ per day, both significantly lower than the NOAEL doses. However, the therapeutic dose of CBG required for treating various conditions currently under investigation might be significantly higher. Table 2 ▸ shows the calculated maximum doses of CBG based on the toxicity levels of the coformers. They are extremely high (over 6 and 14 g per day). Such a high, or even higher, dosing of CBG is unlikely, so we can confidently say that PIP and TMP would not pose toxicity risks as cocrystal formers of CBG.

#### 3.2.5. Crystal structures

Single-crystal XRD was used to fully describe the crystalline structures of pure CBG and the two new cocrystals. The structure of CBG I is in the same crystal form as UHIHEB (Allen, 2002 ▸). But since that was a private communication in the CSD without a proper description and discussion of the structure, here we offer our redetermination at 95 K, together with its comparison with our two cocrystal structures. The selected structural parameters are shown in Table 3 ▸. All other details about the measurement and refinement, as well as the geometry and hydrogen-bonding tables can be found in Tables S6–S12. Figures depicting the asymmetric unit with the thermal ellipsoids, the unit cell with highlighted coformer molecules and hydrogen-bonding patterns are shown in Fig. 6 ▸.

CBG I crystallizes in the orthorhombic system in the space group *P*2_1_2_1_2_1_. There is one molecule of CBG in the asymmetric unit and four of them in the unit cell. The HBs run in the *a* direction, making two parallel infinite chains of ⋯OH⋯OH. The structure is composed of distinct hydro­philic and hydro­phobic layers that alternate parallel to the *ab* plane. The calculated crystal shape filled with molecules shows that the largest faces of the crystal, (002) and (002), have the aliphatic chains of the CBG molecule on the surface, partially explaining the poor aqueous solubility of CBG. The crystals are thin needles, which in turn explains the strong propensity to (001) preferential orientation when examined by XRPD (shown in Fig. S1). When this is taken into account, the calculated XRPD pattern from the structure matches the experimental ones.

CBG–PIP crystallizes in the monoclinic system in the space group *P*2_1_/*n*. There is one molecule of CBG and one molecule of PIP in the asymmetric unit and four of each in the unit cell, thus confirming the 1:1 ratio established using NMR spectroscopy. The HBs run perpendicular to the *b* direction, making an infinite chain of –CBG–PIP–CBG connected by OH⋯N HBs. The structure is composed of distinct hydro­philic and hydro­phobic layers that alternate parallel to the *ac* plane. The hydro­philic layers are much thinner than those of CBG I. The crystal shape of CBG–PIP is a thick plate. The largest faces of the crystal, (020) and (020), have the aliphatic chains of the CBG molecule on the surface, the same as in CBG I, which corresponds to the low IDR improvement. The experimental XRPD pattern matches the one calculated from the structure (see Fig. 2 ▸), confirming the identity and composition of this phase. The visual differences between the calculated and experimental XRPD patterns of CBG–PIP are caused by the preferential orientation of the experimental sample (010).

The structure of CBG–TMP I was successfully solved from single-crystal data. Attempts to create crystals of the CBG–TMP cocrystal forms II and III by slow evaporation of the solvent resulted only in crystals of form I, even when the sample was seeded with particles of forms II and III from previous experiments. We have also tried seeding saturated solutions of CBG and TMP with a few particles of samples from previous experiments on a needle tip. Nevertheless, SCXRD showed that all the resulting crystals were form I. This suggests that form I might be the preferred polymorphic form of the CBG–TMP cocrystal. Our attempts to solve the structures of CBG–TMP II and III from powder data have failed.

**Figure 3:**
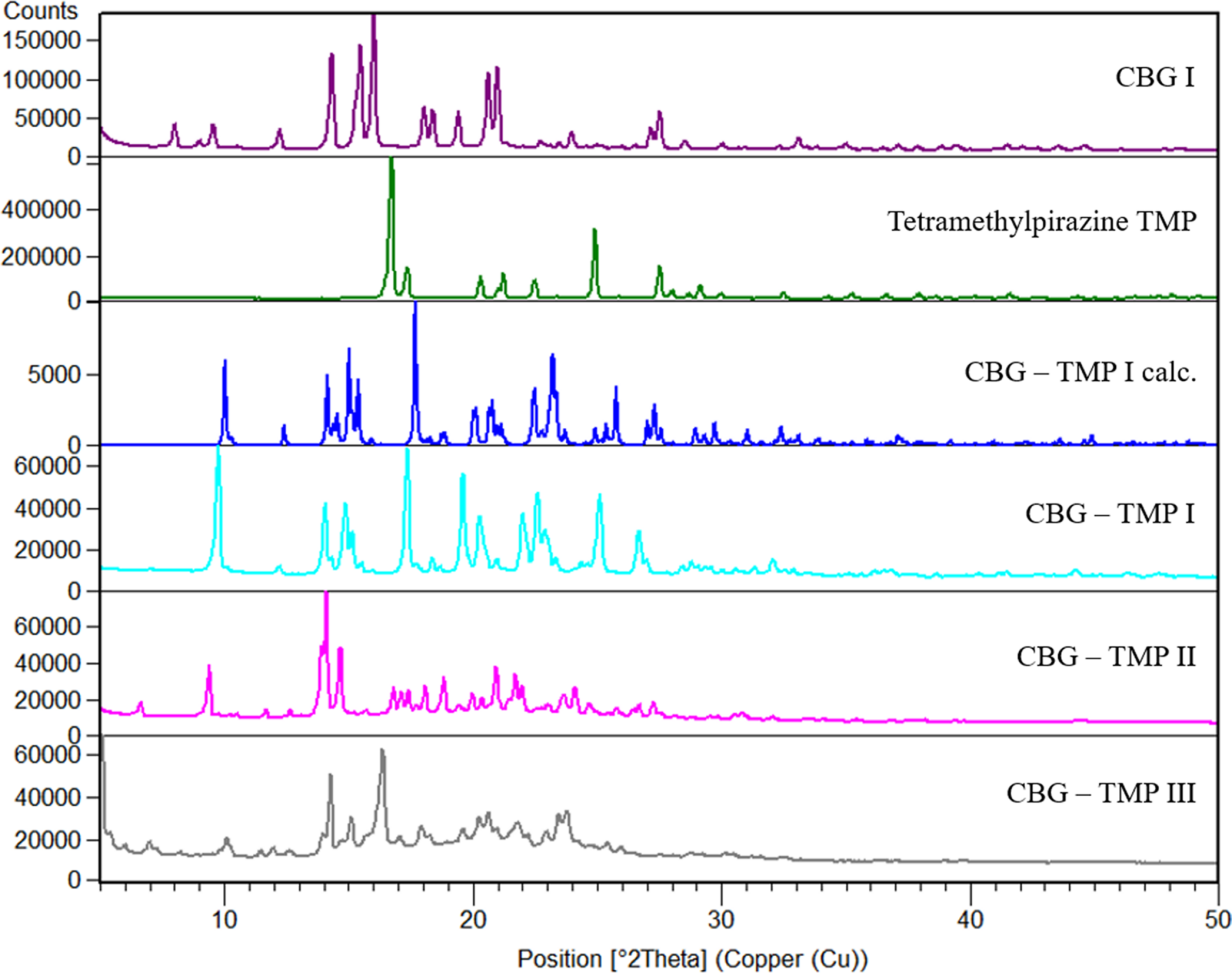
Comparison of powder diffractograms of CBG, TMP and CBG–TMP forms.

**Figure 4:**
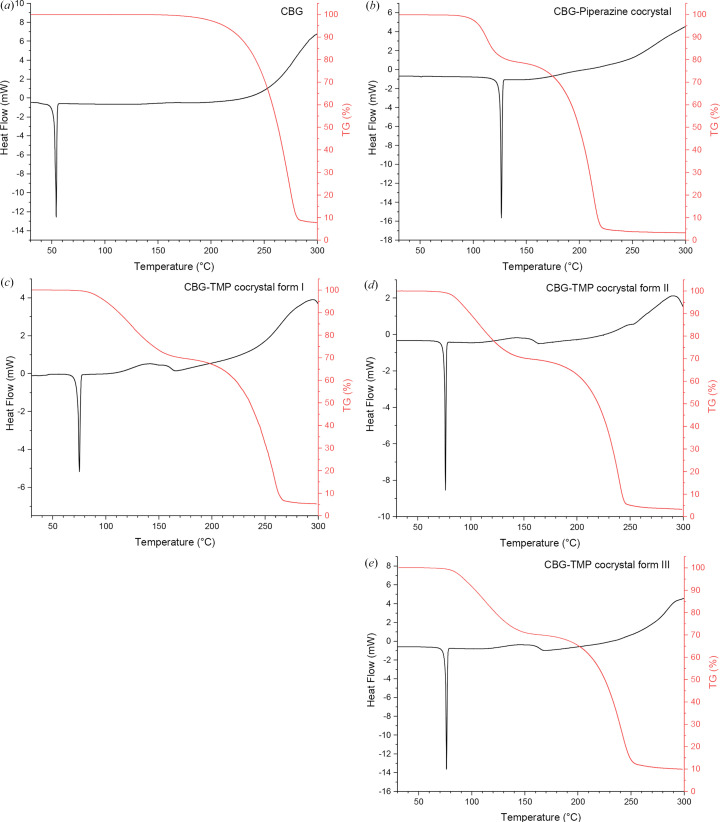
DSC (black) and TGA (red) curves of (*a*) untreated CBG; (*b*) CBG–PIP; and CBG–TMP cocrystal forms (*c*) I, (*d*) II and (*e*) III.

**Figure 5:**
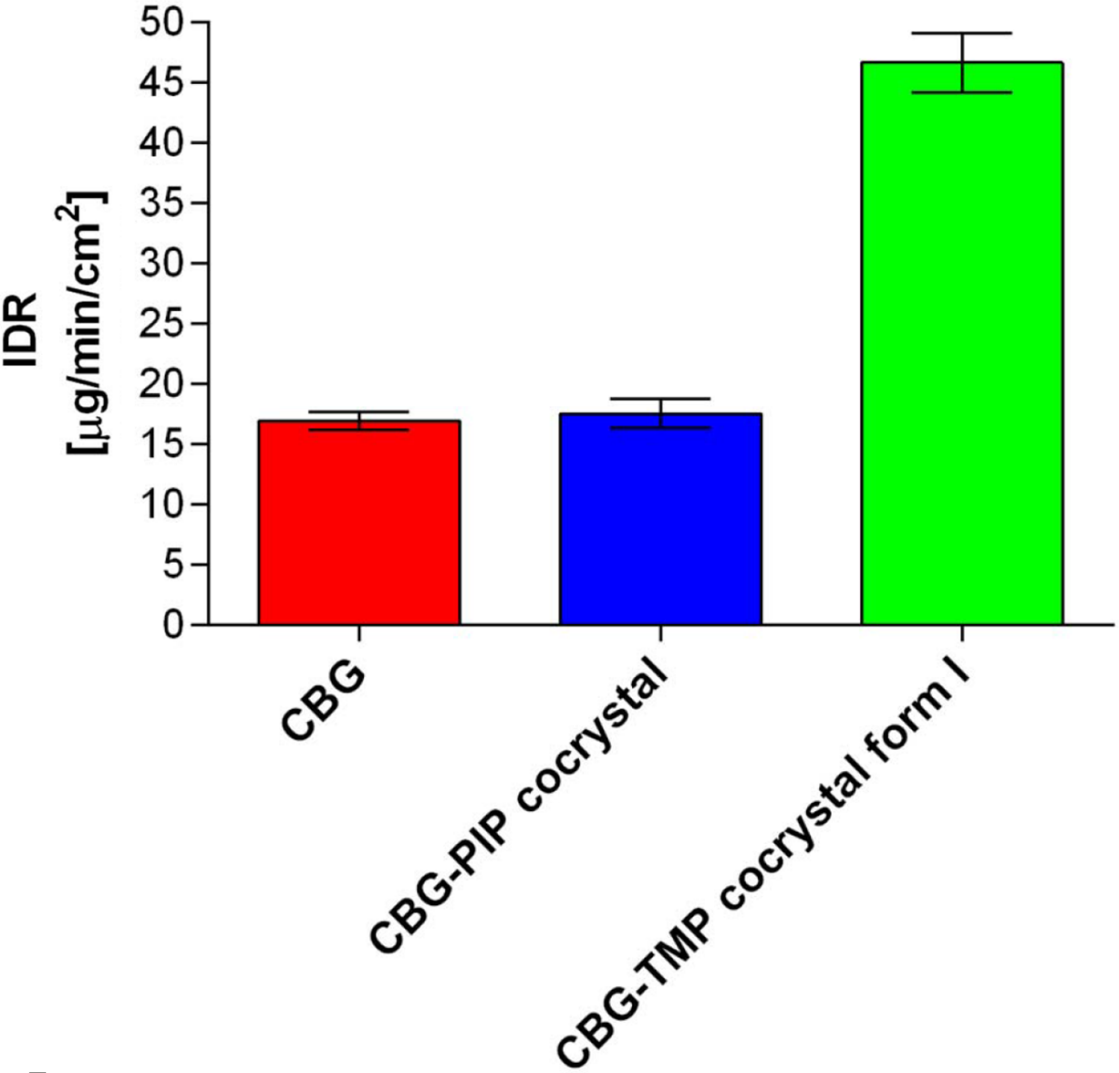
Intrinsic dissolution rate values of CBG and both cocrystals.

**Figure 6:**
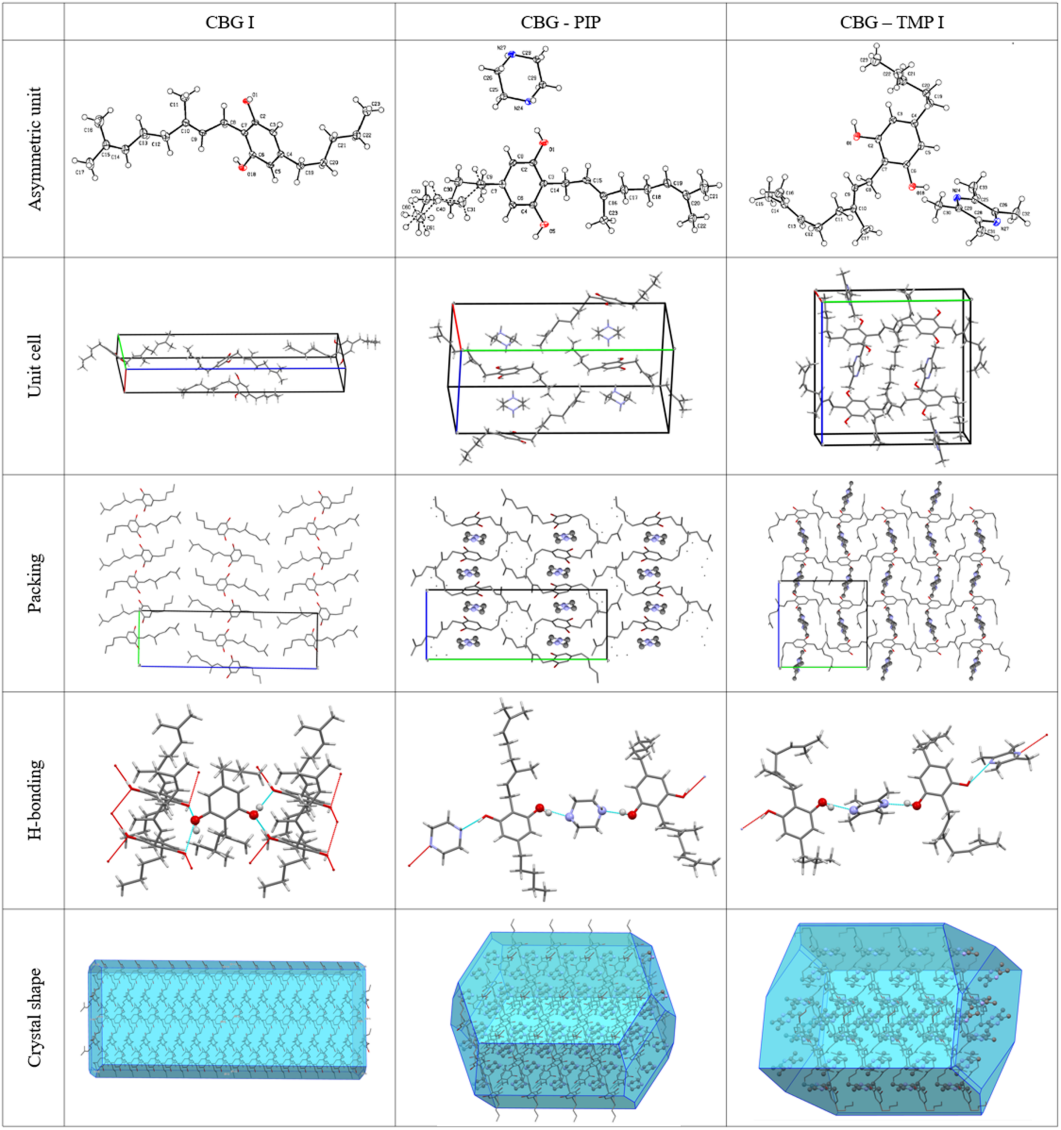
Crystal structures of CBG.

**Figure 7:**
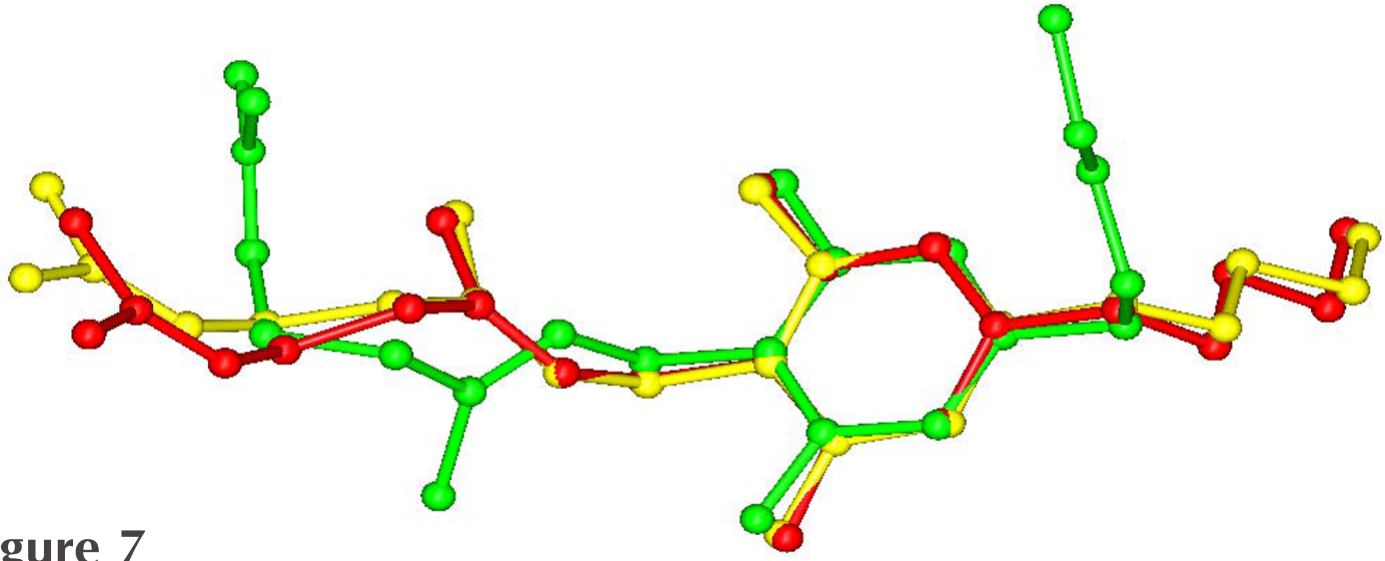
Conformations of CBG in CBG I (red), CBG–PIP (yellow) and CBG–TMP I (green).

CBG–TMP I crystallizes in the monoclinic system in the space group *P*2_1_/*c*. There is one molecule of CBG and one molecule of TMP in the asymmetric unit and four of each in the unit cell, thus confirming the 1:1 ratio established by NMR spectroscopy. The experimental XRPD pattern matches the one calculated from the structure (see Fig. 3 ▸), confirming the identity and composition of this phase. The HBs run perpendicular to the *b* direction, making an infinite chain of –CBG–TMP–CBG connected by OH⋯N HBs. The structure is composed of distinct hydro­philic and hydro­phobic layers that alternate parallel to the *ac* plane. The hydro­philic layers are even thinner than in CBG–PIP, because the aliphatic chains in CBG have a bent conformation, whereas in CBG I and CBG–PIP (see Fig. 7 ▸) they are straight. The crystal shape of CBG–TMP I is a block with facets that have a fairly equal surface. Almost all crystal facets exhibit some level of hydrogen bonding.

#### 3.2.6. Particle surface analysis

**Figure 8:**
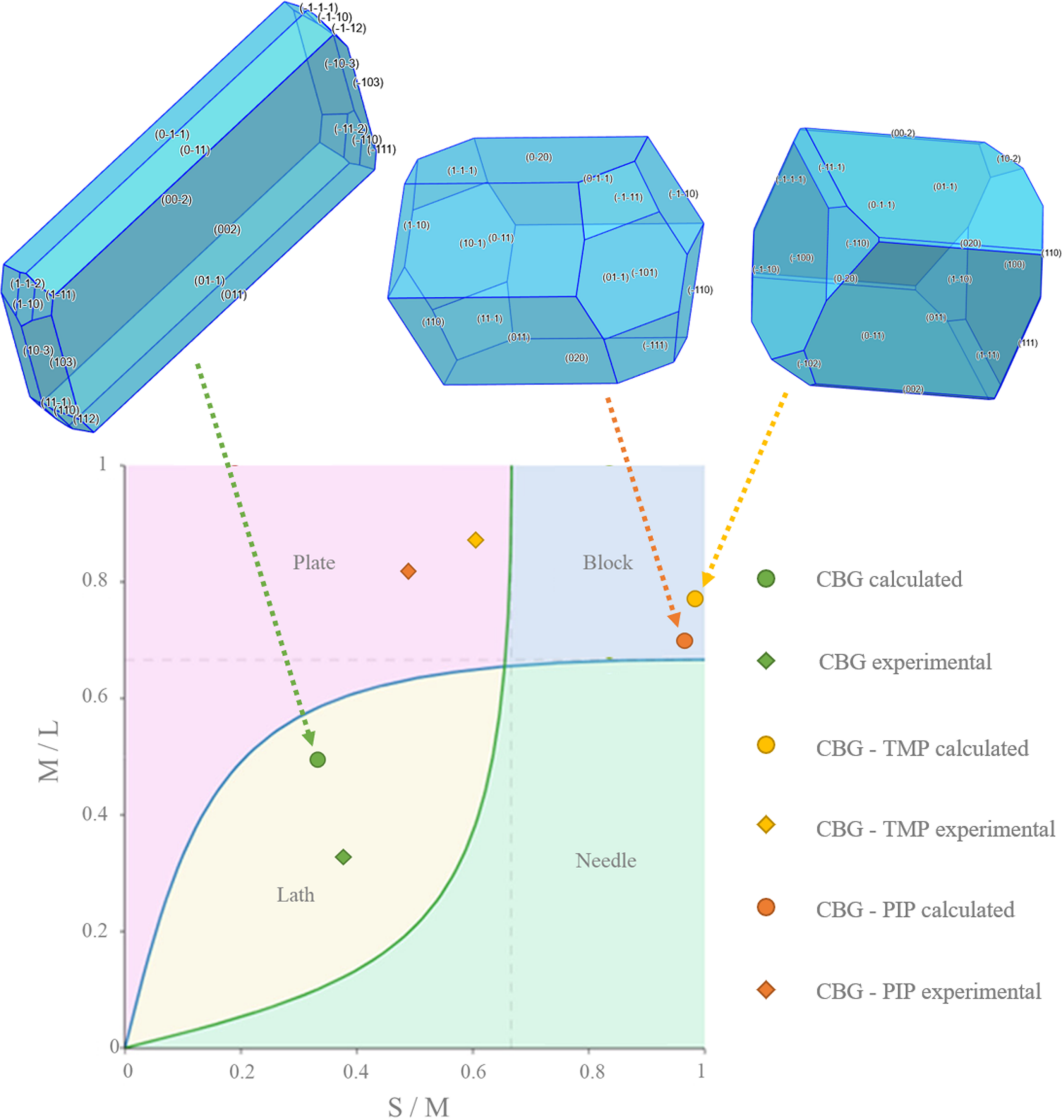
Crystal habits of CBG and its cocrystals calculated by *VisualHabit* compared with the experimental ones using the Zingg plot.

In order to rationalize the bulk properties of the CBG solid forms, especially the dissolution, we performed a comprehensive analysis of the structures using the *CSD-Particle* (Moldovan & Maloney, 2024 ▸) suite in *Mercury*. This new functionality allows for the modelling of the theoretical crystal, assessing its lattice energy as well as its different crystal surfaces. First, the model of the crystal was calculated by the software *VisualHabit*, which provided the lattice energy (see Table S13) and the energies of the intermolecular interactions (synthons), and based on these, the software creates a more realistic crystal habit than just the basic one by the well known Bravais–Friedel–Donnay–Harker (BFDH) method (Clydesdale *et al.*, 1991 ▸). The crystal habits calculated by *VisualHabit* and those experimentally recorded during the SCXRD measurements were compared and analysed using the Zingg plot (Figs. 8 ▸, S10 and Table S14 show the experimental crystal photos and all of the crystal dimensions). The Zingg plot compares the ratios of the crystal dimensions: S – smallest, M – medium, L – largest. By plotting M/L over S/M we show whether the crystal is square (block habit), flat (plate), elongated (needle), or flat and elongated (lath/blade).

The calculated and experimental crystal habits plotted in a Zingg diagram are shown in Fig. 8 ▸. Pure CBG has a lath habit (sometimes also called the blade habit), indicated by values for both the experimental and the theoretical crystal. Both of the cocrystals are significantly more square-shaped than pure CBG. Although the predicted habits were blocks, experimentally, both cocrystals resembled rather thick plates. CBG–TMP is more square whereas CBG–PIP is flatter and more elongated, which is true for both the calculated shapes as well as the experimental ones. The differences between the predicted and observed habits most likely stem from the effect of the solvent on the growth rates of different crystal faces.

**Figure 9:**
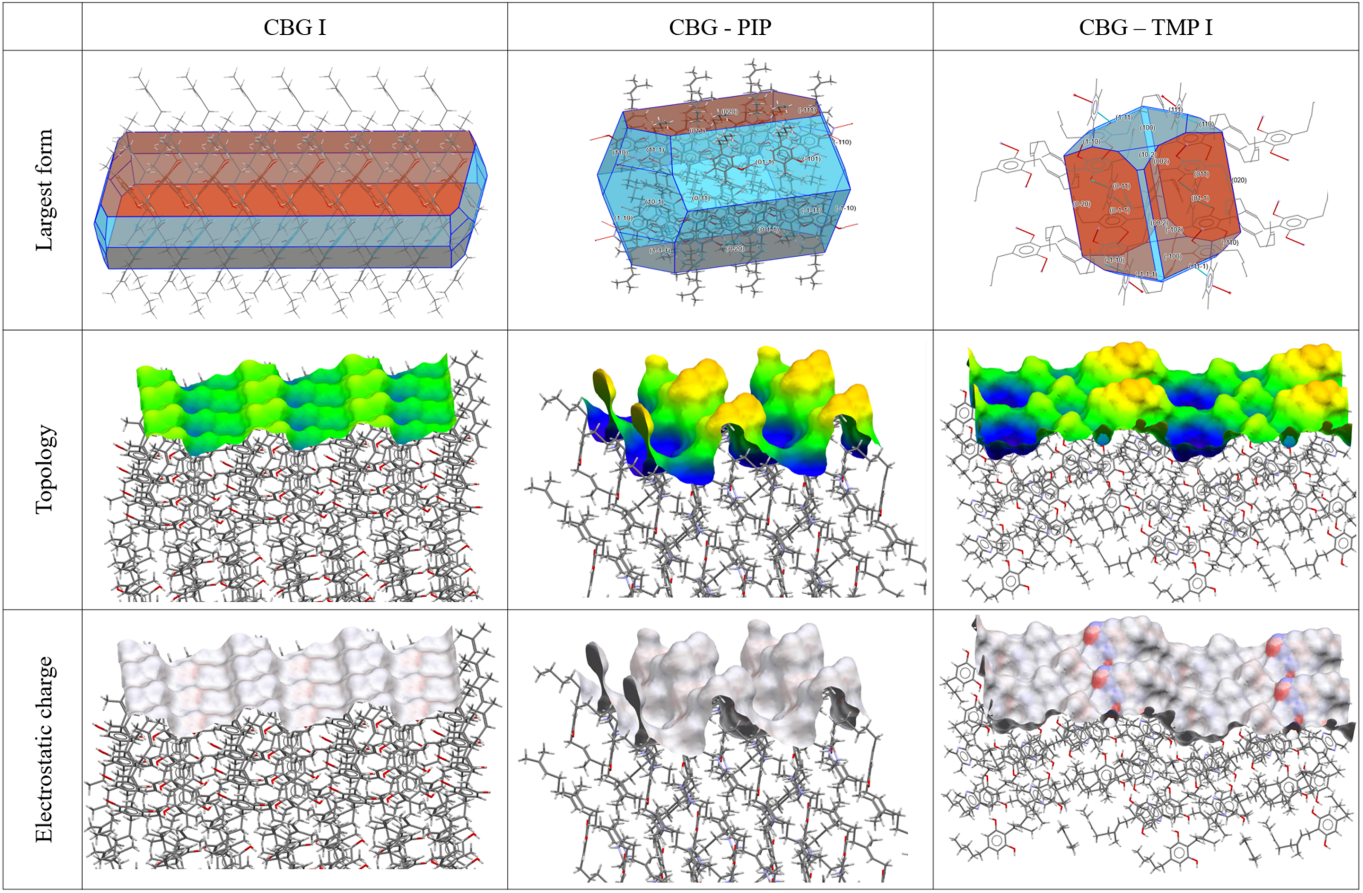
Surface analysis of the largest crystal facets of CBG and its cocrystals. Top – crystal habit filled with molecules showing in which directions the HBs run, and the largest faces (forms) highlighted; middle – topology; bottom – electrostatic charge (red negative, blue positive).

To understand which parameters influence the dissolution properties, we decided to focus on the largest facets of the crystals, as they would have the greatest interaction with the dissolution medium. Based on percentage facet area of all equivalent faces (‘forms’), {002}, {020} and {011} were analysed for CBG I, CBG–PIP and CBG–TMP I, respectively. Table 4 ▸ and Fig. 9 ▸ show the results. Selected parameters considered relevant to dissolution were the attachment energy; rugosity (roughness of the surface); electrostatic Gasteiger charge; the density of HB acceptors, donors and unsatisfied donors; and the water oxygen full interaction maps on the surface (FIMoS) maximum range. The correlations of these parameters to IDRs are shown in Figs. 11 and S11.

*Attachment energy*. In general, crystal faces with lower attachment energy should dissolve faster because less energy is required to detach molecules from these surfaces. However, this was not the case for our system. If a trend had to be identified, it would rather be the opposite.

*Rugosity*. Rougher surfaces (higher rugosity) can increase the surface area in contact with the solvent, potentially enhancing the dissolution rate. Figures for this property are shown in the middle row of Fig. 9 ▸ and numerical values are given in Table 4 ▸. The CBG phase with the highest rugosity by far is CBG–PIP, whereas CBG I and CBG–TMP I are both significantly smoother. No correlation with IDR was observed in our system.

*HB acceptor and donor concentration*. Faces with a higher count of unsatisfied HB donors might dissolve faster because these sites can readily interact with solvent molecules. Similarly, a higher count of HB acceptors can facilitate solvent interactions, potentially increasing the dissolution rate. CBG–TMP I is the only structure that exhibits hydrogen bonding on the largest surface (see Fig. 9 ▸), and it does dissolve significantly faster than the others. So it seems that this category of parameters is very important. Unfortunately, a reasonable correlation cannot be obtained in this case, because for CBG I and CBG–PIP all of these values are 0. Intuitively, we expect the best indicator of this group to be the concentration of the unsatisfied HB donors on the surface because those would be very likely to interact with nearby water molecules.

*Electrostatic charge*. Gasteiger charge provides information about the polarity of functional groups on the crystal surface. In the cases of CBG I and CBG–PIP, the colours indicating the electrostatic charge are very pale, almost white, because the surface consists of aliphatic chains. The main surface of CBG–TMP I contains hydroxyl groups that would be involved in hydrogen bonding; these are the red (HB acceptor) and blue (HB donor) areas on the electrostatic charge surface map. The charged areas are where polar interactions with water would be expected. The same is reflected by the numerical values. The difference between the values of charges of the most positive and most negative atoms on the surface (Table 4 ▸) show a near-perfect correlation when plotted against the IDRs (Fig. 11).

**Figure 10:**
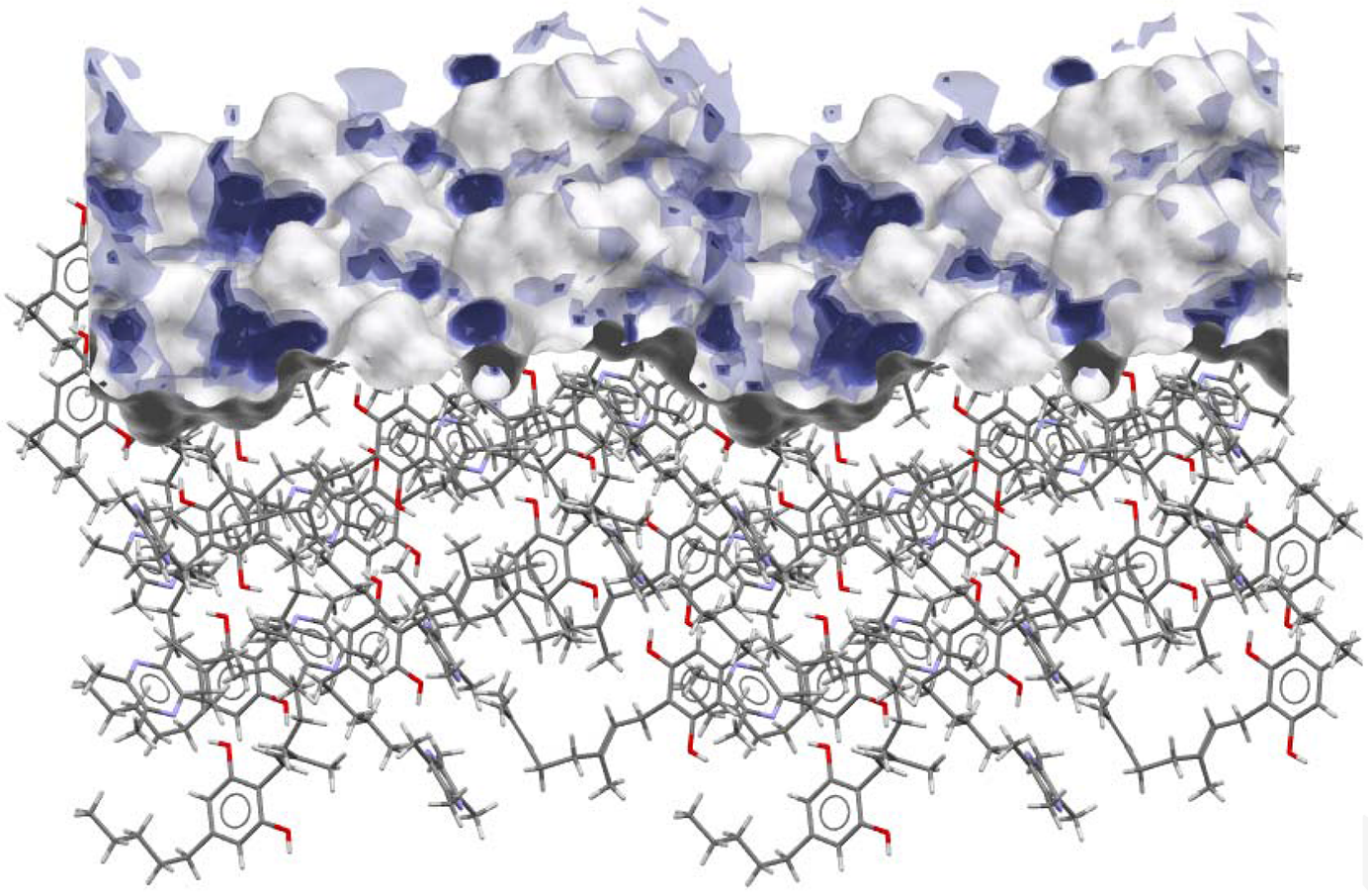
Full interaction maps generated for the (011) surface of CBG–TMP I showing the likely interaction sites for water molecules.

**Figure 11:**
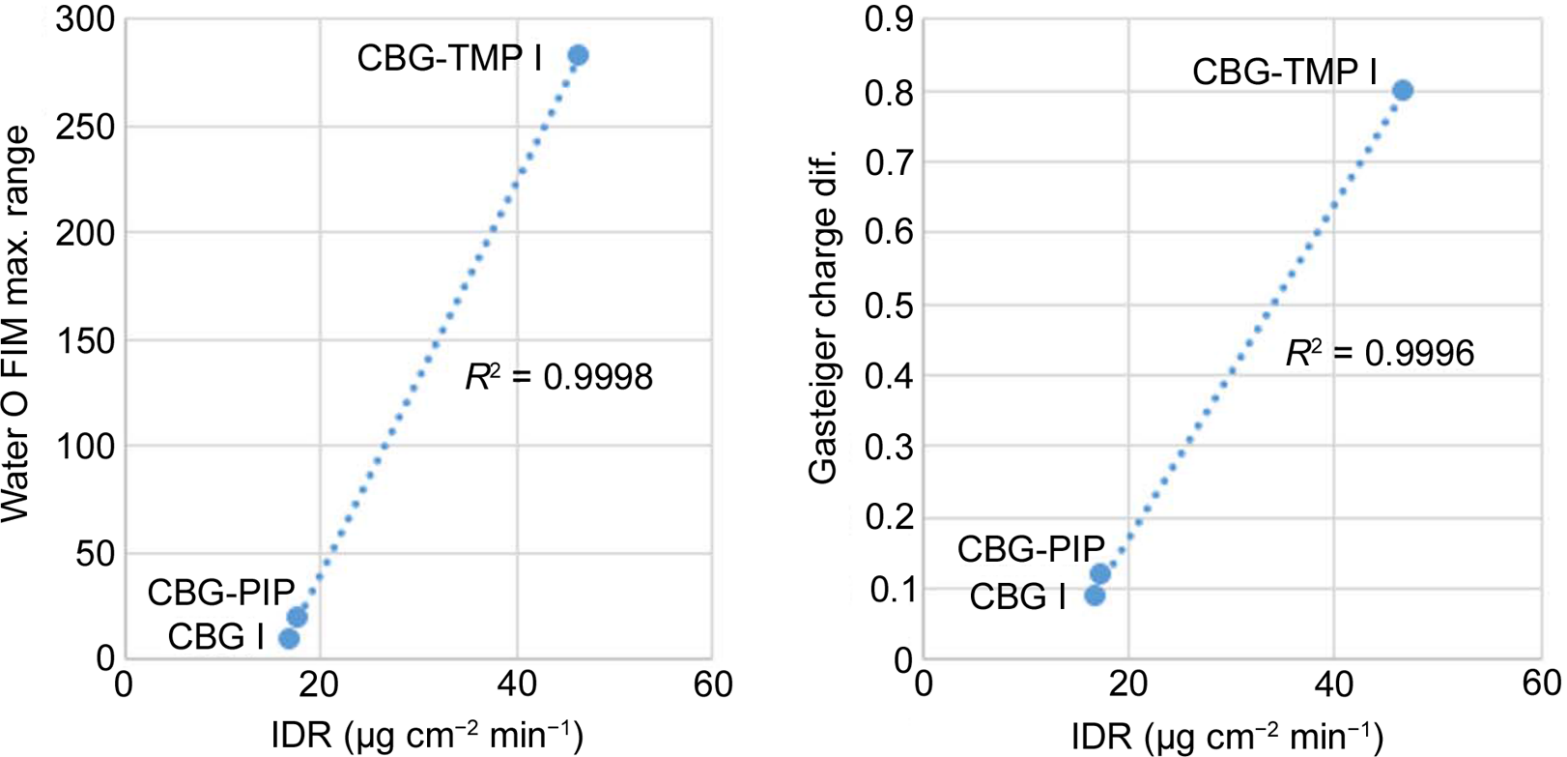
Water FIMoS (calculated for the water probe) maximum range for CBG and its cocrystals (left) and the electrostatic charge difference (right) plotted against the IDRs.

*Water FIMoS maximum range*. FIMoS predict where interactions are most likely to occur on the crystal surface (Fig. 10 ▸). Crystal faces with a higher water FIMoS maximum range are more likely to form hydration shells, which facilitate the dissolution process by stabilizing detached molecules in the solvent. Water FIMoS maximum range values for CBG and its cocrystals are shown in Table 4 ▸, with a near-perfect correlation when plotted against the IDRs (Fig. 11 ▸).

## 4. Conclusions

In this study, we investigated the solid forms of the natural compound CBG to enhance its physicochemical properties, linking crystal structures to these bulk properties through particle analysis. Our cocrystal screening identified two promising new cocrystals: one with piperazine and another with tetra­methyl­pirazine. The cocrystal with tetra­methyl­pirazine was particularly noteworthy due to its existence in three polymorphic forms. Both cocrystals demonstrated improved melting points, with the tetra­methyl­pirazine cocrystal also exhibiting a significant enhancement in dissolution rate. Unlike the pure CBG with its lath habit, both cocrystals had crystal habits more suitable for pharmaceutical processing. A comparative crystal structures analysis revealed the structural basis for the observed improvements in physicochemical properties. To identify properties linked to dissolution, we used the *CSD-Particle* suite to analyse crystal morphologies and surfaces. Although surface attachment energy and roughness (rugosity) did not show significant effects, the concentration of unsatisfied HB donors displayed a positive correlation with dissolution rate. Notably, two parameters showed a very strong correlation with dissolution rate: the propensity for interactions with water molecules, determined by the maximum range in the full interaction maps on the surface calculated for the water probe, and the difference in positive and negative electrostatic charges. These parameters demonstrated strong predictive capability for aqueous dissolution. Further testing on additional systems is needed to confirm their universal applicability, but these parameters hold promise for significantly enhancing the predictability of aqueous dissolution processes. Combined with the increasing availability and reliability of crystal structure prediction, this approach could streamline pharmaceutical development efforts by focusing on materials with desired predicted properties.

## Supplementary Material

Crystal structure: contains datablock(s) global, CBG_TMP, CBG_PIP, CBG_I. DOI: 10.1107/S2052252525001009/lt5073sup1.cif

Structure factors: contains datablock(s) CBG_PIP. DOI: 10.1107/S2052252525001009/lt5073CBG_PIPsup2.hkl

Structure factors: contains datablock(s) CBG_I. DOI: 10.1107/S2052252525001009/lt5073CBG_Isup3.hkl

Structure factors: contains datablock(s) CBG_TMP. DOI: 10.1107/S2052252525001009/lt5073CBG_TMPsup4.hkl

Supporting figures and tables. DOI: 10.1107/S2052252525001009/lt5073sup5.pdf

CCDC references: 2390061, 2390062, 2390063

## Figures and Tables

**Table 1:** Coformers used for cocrystal screening

Coformer	Abbreviation	Selection criteria†
4-methyl­pyridin-*N*-oxide	4X	AP, CA
Arginine	AR	ZW, AA
Caffeine	CAF	CA
Glutamic acid	GA	ZW, AA
Glutamine	GL	ZW, AA
Hippuric acid	HU	AA
Indole	ID	CA
Isonicotinamide	IN	AP, AA, CA
Isonicotinic acid *N*-oxide	IX	AP, AA, CA
Lidocaine	LD	AA
Lysine	LY	ZW, AA
Nicotinamide	NI	AP, AA, CA
Piperazine	PIP	CA
Polydatin (Piceid)	PL	CA
Pyridine-*N*-oxide	PX	AP, CA
Quercetin	QE	CA
Tetra­methyl­pyrazine	TMP	KC, CA
Tryptophan	TR	AA, CA, ZW
Valine	VA	ZW, AA

† AP: alcohol–pyridine/pyridine-*N*-oxide synthon; CA: cyclic/aromatic compound with nitro­gen in the ring; ZW: zwitterionic compound; AA: alcohol–amide synthon; KC: known to form a cocrystal with cannabidiol. See *Results*[Sec sec3] for further discussion.

**Table 2:** Safety notes on the CBG coformers

Coformer	NOAEL (mg kg^−1^ body weight per day)	Safe dose† (mg per day)	Corresponding dose of CBG (mg per day)
PIP	25‡	1750	6440
TMP	55 (Adams *et al.*, 2002 ▸)	3850	14169

† For a 70 kg person.

‡ European Union Risk Assessment Report (https://echa.europa.eu/documents/10162/35f9602c-cb84-448f-9383-250e1a5ad350).

**Table 3:** Selected crystallographic data for CBG structures

Crystal data	CBG I	CBG–PIP	CBG–TMP I
CBG:coformer ratio	–	1:1	1:1
Crystal system	Orthorhombic	Monoclinic	Monoclinic
Space group	*P*2_1_2_1_2_1_	*P*2_1_/*n*	*P*2_1_/*c*
*a* (Å)	4.5073 (1)	8.7047 (1)	8.8766 (1)
*b* (Å)	11.4901 (1)	26.7487 (1)	17.6450 (1)
*c* (Å)	36.7494 (3)	11.0627 (1)	17.2008 (1)
α, β, γ (°)	90, 90, 90	90, 111.2361 (8), 90	90, 91.5518 (6), 90
*V* (Å^3^)	1903 ▸.23 (5)	2400.92 (4)	2693.13 (4)

**Table 4:** Selected surface characteristics for the largest crystal forms of CBG and its cocrystals Form – a set of crystallographically equivalent faces.

	CBG I	CBG–PIP	CBG–TMP I
Miller indices	{002}	{020}	{011}
Percentage facet area (all equiv. faces)	65.804	25.918	65.1
Attachment energy (kJ mol^−1^)	−22.198	−34.514	−43.642
Rugosity	1.331	2.326	1.761
Gasteiger charge max.	0.03	0.04	0.29
Gasteiger charge min.	−0.06	−0.08	−0.51
Gasteiger charge diff.	0.09	0.12	0.8
HB acceptors (count/Å^2^)	0	0	0.018
HB donors (count/Å^2^)	0	0	0.009
HB donors unsatisfied (count/Å^2^)	0	0	0.005
Water O FIMoS max. range	9.28	19.54	282.86
