# Supplementary material for: Surface analysis of cannabigerol cocrystals: linking crystal structure to enhanced properties
Source: IUCrJ. 2025 Feb 27;12(Pt 2):141–54. doi: 10.1107/S2052252525001009 (PMC11878449; doi:10.1107/S2052252525001009)
Supplement: Supplementary file 5 [file m-12-00141-sup5.pdf]

IUCrJ

Volume 12 (2025)

Supporting information for article:

Surface analysis of cannabigerol cocrystals: linking crystal structure to enhanced properties

Eliška Zmeškalová, František Stara, Tereza Havlůjová and Miroslav Šoóš

Polymorph and solvate screening

For the polymorph and solvate screening, sixteen samples were prepared using the slow evaporation method, and one sample was prepared using liquid-assisted grinding. The approximate solubilities are presented in Table S1.

Table S1 – Solvents, their distributors, and properties.

Solvent	Abbreviation	Distributor	Purity
Acetone	AE	Lach-Ner, s.r.o.	G.R., ISO reagent
Ethyl acetate	EA	Lach-Ner, s.r.o.	G.R.
Methanol	ME	Lach-Ner, s.r.o.	p.a., ISO reagent
Ethanol	ET	Lach-Ner, s.r.o.	96% G.R.
Toluene	TO	PENTA s.r.o.	p.a.
Dichloromethane	DC	PENTA s.r.o.	p.a.
Diethyl ether	DE	Lach-Ner, s.r.o.	G.R.
Acetonitrile	AN	Lach-Ner, s.r.o.	G.R.
Tetrahydrofuran	TH	Lach-Ner, s.r.o.	G.R.
Heptane	HP	PENTA s.r.o.	99% p.a.
Hexane	HX	Lach-Ner, s.r.o.	G.R.
Cyclohexane	CHX	Lach-Ner, s.r.o.	G.R.
Benzene	BZ	Lach-Ner, s.r.o.	G.R.
Dioxane	DX	PENTA s.r.o.	p.a.

Table S2 – Used solvent mixtures and their details.

Solvent : Solvent	Volumetric ratio	Abbreviation
Heptane:Ethanol	95:5	HPET
Hexane:Ethanol	95:5	HXET

To determine if the experiments produce new solid forms, the powder diffractograms of the samples were compared to the diffraction pattern of CBG I calculated from its crystal structure, shown in Figure . All sample XRPDs are shown in Figure S2 in the Supporting information. The experiments did not produce new solid forms – all the samples exhibited crystallinity and remained in the starting solid form CBG I. The theoretical powder diffraction pattern of the CBG crystal structure matches the experimental patterns, even though there are visual differences between the experimental XRPD patterns. However, these are

only caused by a strong preferential orientation (001 direction) in some of the samples. This can be better understood by looking at the calculated shape of the crystal shown in Figure 9. The crystals are long, thin needles and, therefore, prone to preferential orientation.

Table S3 – Polymorph and solvate screening experiments of CBG by slow evaporation

Solvent	Approximate solubility [g/l]	XRPD solid form
Acetone	250.0	CBG I
Acetonitrile	1227.5	CBG I
Benzene	2360.0	CBG I
Cyclohexane	86.4	CBG I
Dichloromethane	252.0	CBG I
Diethyl ether	125.8	CBG I
Dioxane	259.0	CBG I
Ethyl acetate	1297.5	CBG I
Ethanol	125.3	CBG I
Heptane	4.5	CBG I
Hexane	8.9	CBG I
Methanol	100.6	CBG I
Tetrahydrofuran	2540.0	CBG I
Toluene	31.7	CBG I
Heptane–Ethanol (95:5)	83.7	CBG I
Hexane–Ethanol (95:5)	214.4	CBG I

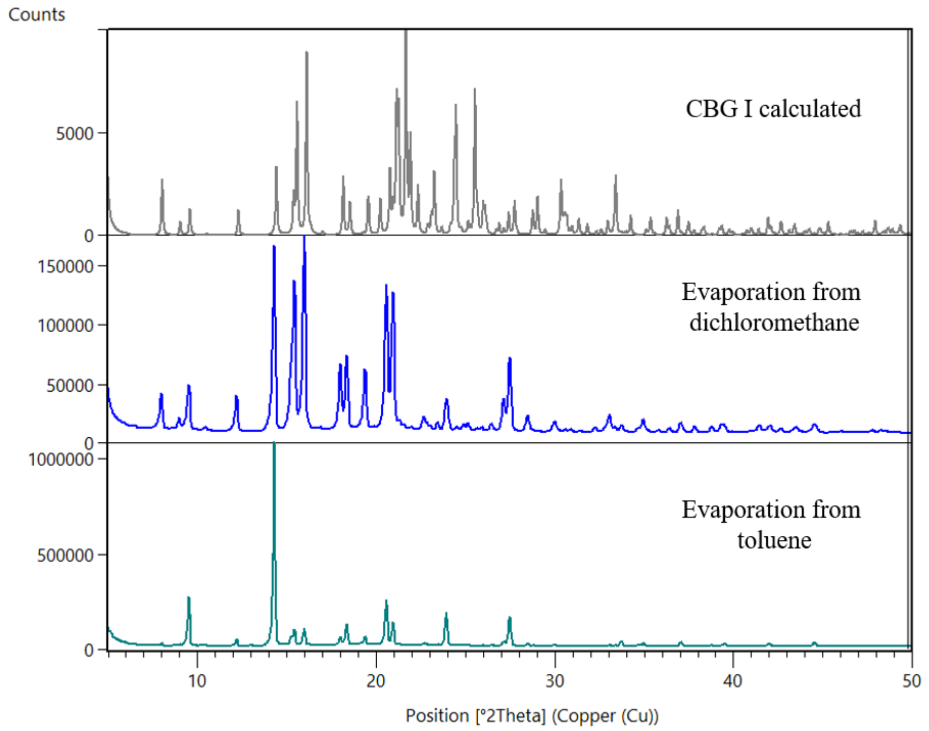

Figure S1 – Examples of powder diffractograms of CBG samples after polymorph and solvate screening, compared to the calculated one from the crystal structure of CBG I. The sample from toluene shows a strong preferential orientation in 001 direction

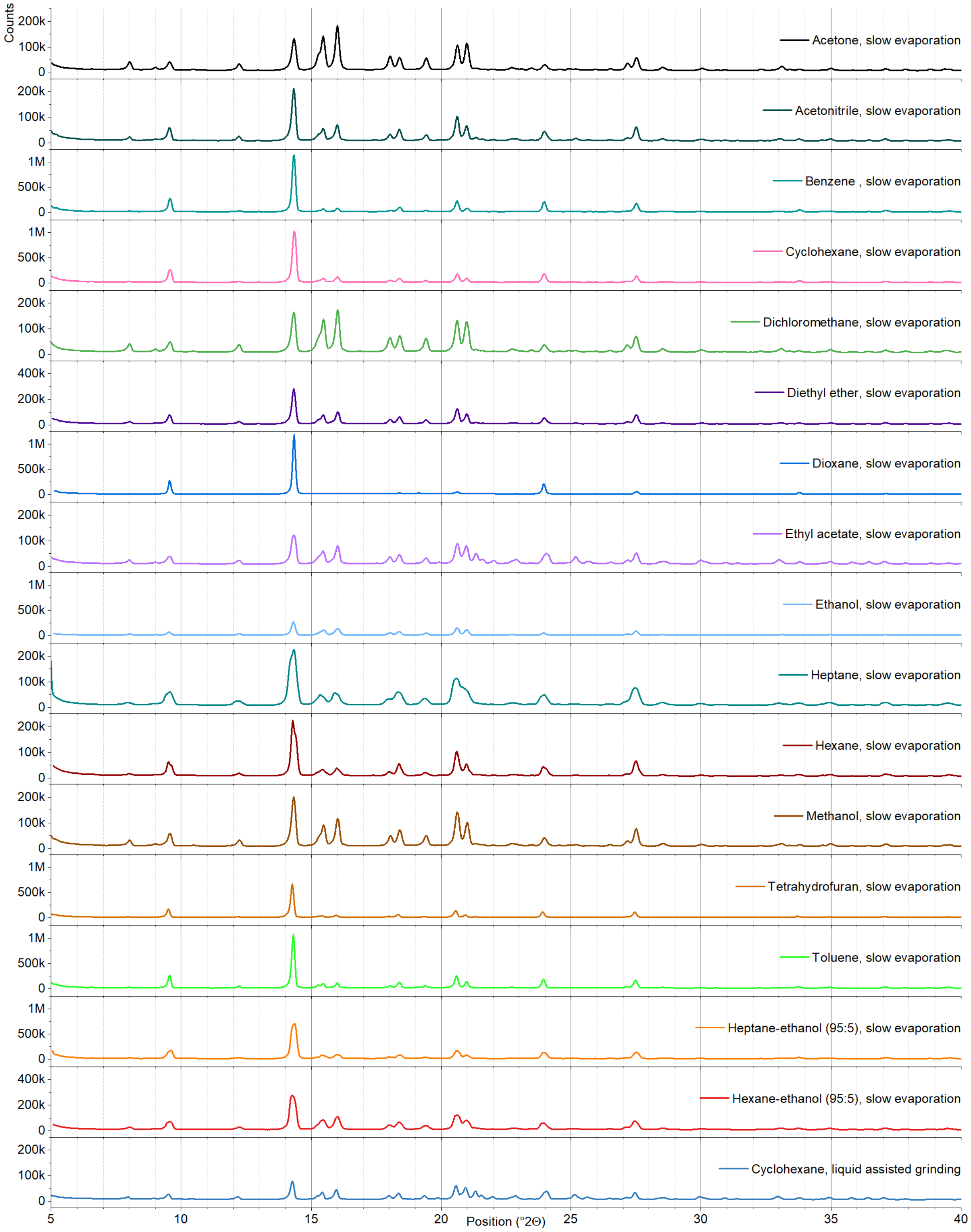

Figure S2 – Powder diffractograms of CBG from polymorph and solvates screening.

Cocrystal screening

The screening setups were slow evaporation from ethanol, slow evaporation or slurry mixing from acetone, slurry mixing from cyclohexane, slurry mixing from a solution of 95:5 heptane–ethanol, and liquid-assisted grinding with cyclohexane. Slow evaporation from ethanol and acetone were chosen because of the high solubility of CBG in both ethanol and acetone. Cases in which the coformer did not dissolve completely in 500 µl of acetone were carried out as slurry mixing instead. Cyclohexane and a mixture of heptane–ethanol (95:5) were chosen for the slurry mixing due to their medium solubility of CBG allowing it to form a suspension. Medium solubility in cyclohexane was also the reason for its use in liquid assisted grinding. The CBG cocrystal screening experiments are summarised in the outcome table – Table S4.

Table S4 – Summary of CBG cocrystal screening experiments. Each cell represents a result of one screening experiment with rows denoting a given coformer and columns denoting solvent and method of preparation. Solvents include ethanol (ET), acetone (AE), cyclohexane (CHX) and a 95:5 mixture of heptane–ethanol (HPET). "DNC" denotes experiment did not crystallise, "sm" denotes experiment resulted in a simple mixture of constituent solid phases and "New phase" denotes experiment resulted in solid phase with an unknown powder diffractogram.

	ET	AE		HPET (95:5)	CHX	
Coformer	Slow evaporation	Slow evaporation	Slurry mixing	Slurry mixing	Slurry mixing	Liquid assisted grinding
4X	DNC	DNC	–	DNC	DNC	DNC
AR	–[1]	–	sm	sm	sm	sm
CAF	sm	–	sm	sm	sm	sm
GA	sm	–	sm	sm	sm	sm
GL	–[2]	–	sm	sm	sm	sm
HU	sm	–	sm	sm	sm	sm
ID	DNC	DNC	–	sm	sm	sm
IN	sm	DNC	–	sm	sm	sm
IX	sm	–	sm	sm	sm	sm
LD	DNC	DNC	–	DNC	DNC	DNC
LY	sm	–	DNC	sm	sm	sm
NI	DNC	DNC	–	sm	sm	sm
PI	**New form**	–	**New form**	**New form**	**New form**	**New form**
PL	sm	–	sm	sm	sm	sm
PX	DNC	DNC	–	DNC	DNC	DNC

[1] Experiment omitted because of very low solubility of arginine in ethanol.
[2] Experiment omitted because of very low solubility of glutamine in ethanol.

	ET	AE		HPET (95:5)	CHX	
Coformer	Slow evaporation	Slow evaporation	Slurry mixing	Slurry mixing	Slurry mixing	Liquid assisted grinding
QE	sm[3]	–	DNC	sm	sm	sm
TMP	**New form I**	**New form I**	–	**New form II**	**New form I**	**New form I**
TR	sm	–	sm	sm	sm	sm
VA	sm	–	sm	sm	sm	sm

There are three types of outcomes observed from these screening experiments. In some experiments, the sample turned into a viscous liquid phase unable to form crystals under ambient conditions; these results are denoted as "did not crystallise" (DNC) in the outcome table. In most experiments where the resulting substance was a solid, the corresponding powder diffractogram showed only a combination of signals from both combined solids – CBG and coformer; these samples represent cases in which no cocrystal formed and the resulting solid was simply a mixture of materials. These results are denoted as "simple mixture" (sm) in the outcome table. In a couple of cases, a new solid form appeared. Powder diffractograms of these solid samples showed peaks from neither the CBG nor the coformer, instead the diffractogram was completely new.

Evidence suggesting the formation of a new phase in CBG–piperazine experiments includes the two strong peaks at positions 6.2° and 12.9° 2θ, the absence of peaks characteristic of CBG at 8.1° and 15.5° 2θ, and the absence of peaks characteristic of piperazine at 18° and 19.6° 2θ as well as the broad background halo signifying an amorphous phase.

Evidence suggesting the formation of a new CBG–TMP phase (denoted as form I) includes peaks at positions 9.6°, 14°, 19.6°, 22°,25.1°, 26.8° 2θ, the absence of CBG peaks at 8.1° and 15.5° 2θ and the absence of TMP peaks at 16.5°, 21.1°, 24.9° and 27.5° 2θ.

Evidence suggesting the formation of a new CBG–TMP phase (denoted as form II) includes peaks at positions 6.8°, 9.9°, 14°, 14.1°,14.8° and 19° 2θ, the absence of CBG peaks at 8.1° and 15.5° 2θ and the absence of TMP peaks at 16.5°, 23.5° and 24.9° 2θ.

[3] Because of poor solubility of quercetine in ethanol, slurry mixing experiment in ethanol was carried out as well. The resulting powder exhibited a distinctive powder diffration pattern suggesting a formation of a new phase. However, NMR spectroscopy of this sample identified the internal stoichiometry as being 2 quercetine : 1 ethanol : 0.1 CBG. With these molar ratios the new phase is more likely a quercetine-ethanol solvate contaminated with a residue of CBG, rather than a CBG-quercetine cocrystal. For this reason, it was not further studied as part of this work.

Crystallization Conditions and Polymorph Identification of CBG-PIP

This section discusses the optimization of crystallization conditions to determine the specific parameters under which the cocrystal forms. It also addresses the identification of the polymorphic forms of the cocrystal, ensuring that the most stable and effective form is selected for further development. During the screening, the CBG–piperazine cocrystals were prepared by slow evaporation of ethanol and ethyl acetate, by slurry mixing in cyclohexane and heptane–ethanol 95:5 solution, and by liquid assisted grinding with cyclohexane. The robustness of preparation of this cocrystal was tested by trying the preparation with modified parameters. The resulting crystalline powders were analysed using XRPD to establish whether the resulting solid phase is the same. Slurry mixing of CBG and piperazine in molar ratios of 1/1, 1/2 and 2/1 resulted in powders of pure cocrystal in cases where the sample was filtered after mixing. In cases where instead of filtration the solvent was simply allowed to evaporate the resulting powder contained the excess component together with the cocrystal. The method of slow evaporation of the solvent was tested using acetone, acetonitrile, dichloromethane, diethyl ether, ethyl acetate, ethanol, heptane, methanol, tetrahydrofuran, and toluene. All of these evaporation experiments resulted in the same new CBG–piperazine cocrystal, except for evaporation of toluene which resulted in an oily substance that did not crystallise.

Crystallization Conditions and Polymorph Identification of CBG-TMP

To assess the robustness of the preparation of the CBG–TMP cocrystal and to discover which parameters have an impact on determining the polymorphic phase, the slow evaporation method was carried out in ten solvents. 20 mg of CBG and an equimolar amount of TMP were dissolved in 500 µl of solvent, and the solutions were left in a fume hood so that the solvent could evaporate. The dried samples were analysed using XRPD and compared with the already discovered forms I and II. The results of these experiments are summarised in Table S5. One additional solid form was discovered – form III resulting from evaporation of diethyl ether. The main peaks that are specific for form III are at positions 5.0° and 16.4° (see Figure 3).

Table S5 – Results of slow evaporation experiments for mixtures of CBG and TMP.

CBG–TMP cocrystal, slow evaporation

Solvent	Molar ratio (CBG:TMP)	$V_{solvent}$ [µl]	XRPD Solid form
Acetone	1:1	500	Form I
Acetonitrile	1:1	500	Form I
Dichloromethane	1:1	500	Form I
Diethyl ether	1:1	500	Form III
Ethyl acetate	1:1	500	Form I

CBG–TMP cocrystal, slow evaporation

Solvent	Molar ratio (CBG:TMP)	$V_{solvent}$ [μl]	XRPD Solid form
Ethanol	1:1	500	Form II
Heptane–ethanol (95:5)	1:1	500	Form I
Methanol	1:1	500	Form I
Tetrahydrofuran	1:1	500	Form II
Toluene	1:1	500	Form I

The experiments that produced the new forms were repeated to test which parameters influenced the formation of a particular solid form. The slow evaporation of acetone produced mostly form I. Slow evaporation of diethyl ether can produce forms I, II, and III, so far without a reliable way to predict the outcome. Slow evaporation of ethanol or tetrahydrofuran usually led to form II and occasionally to form I. Unfortunately, upon scaling the experiments (amount of CBD, TMP and solvent multiplied by 10), samples from diethyl ether, ethanol and tetrahydrofuran repeatedly resulted only in form I. Due to very high solubility in organic solvents of not only CBG and TMP but their cocrystal as well, no seeding procedure for polymorph control seemed feasible. Therefore, evaporation conditions and concentration of solution were considered as possible key parameters for polymorph control. In a series of experiments, we tested the effect of CBG, TMP concentration by dissolving the necessary amount in either 1 or 5 ml of solvent. The effect of evaporation conditions was investigated as follows, the samples of same composition were either left in vials or poured into Petri dishes, one of each was then left at ambient conditions or placed on hot plate heated up to 30°C to evaporate, resulting powders were then analysed by XRPD. However, all samples contained form I, no other polymorph was obtained in sufficient amount for further testing.

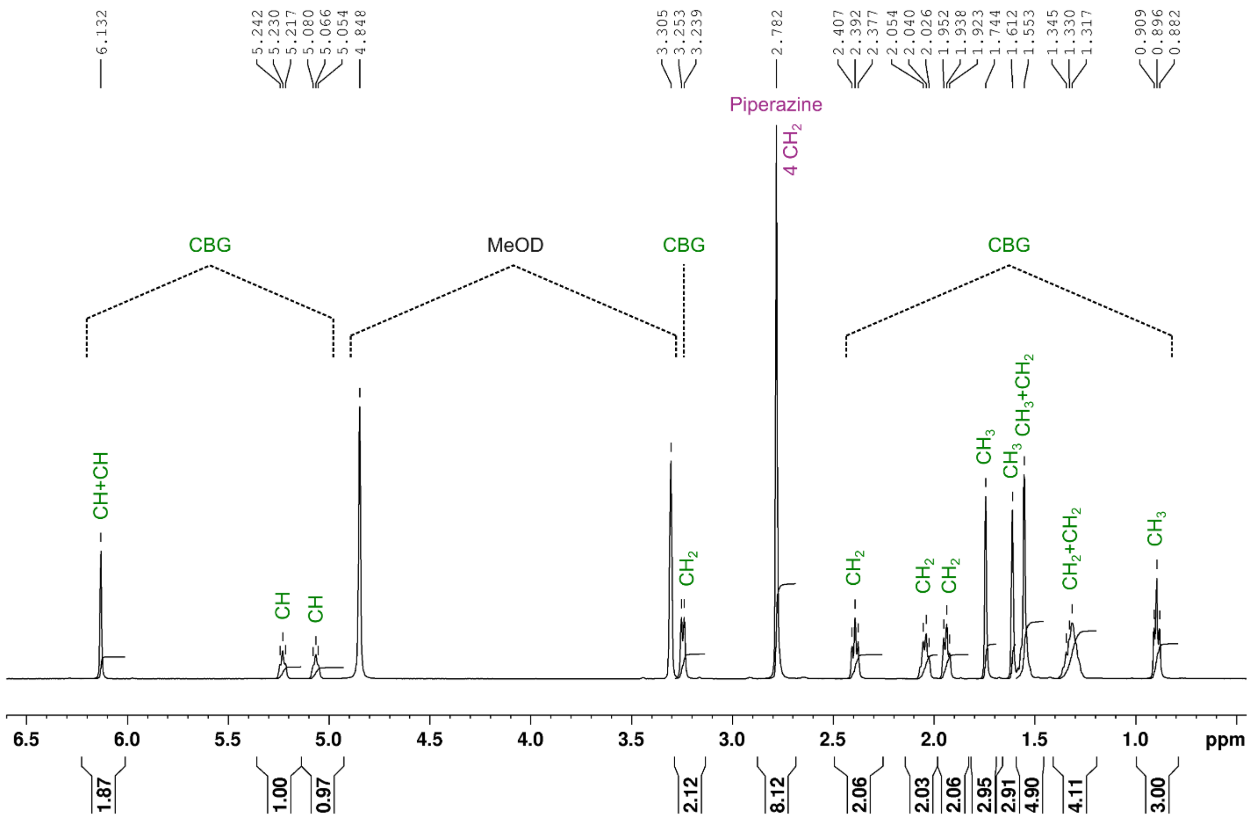

Figure S3 – ^{1}H NMR spectrum of a novel CBG–piperazine solid phase.

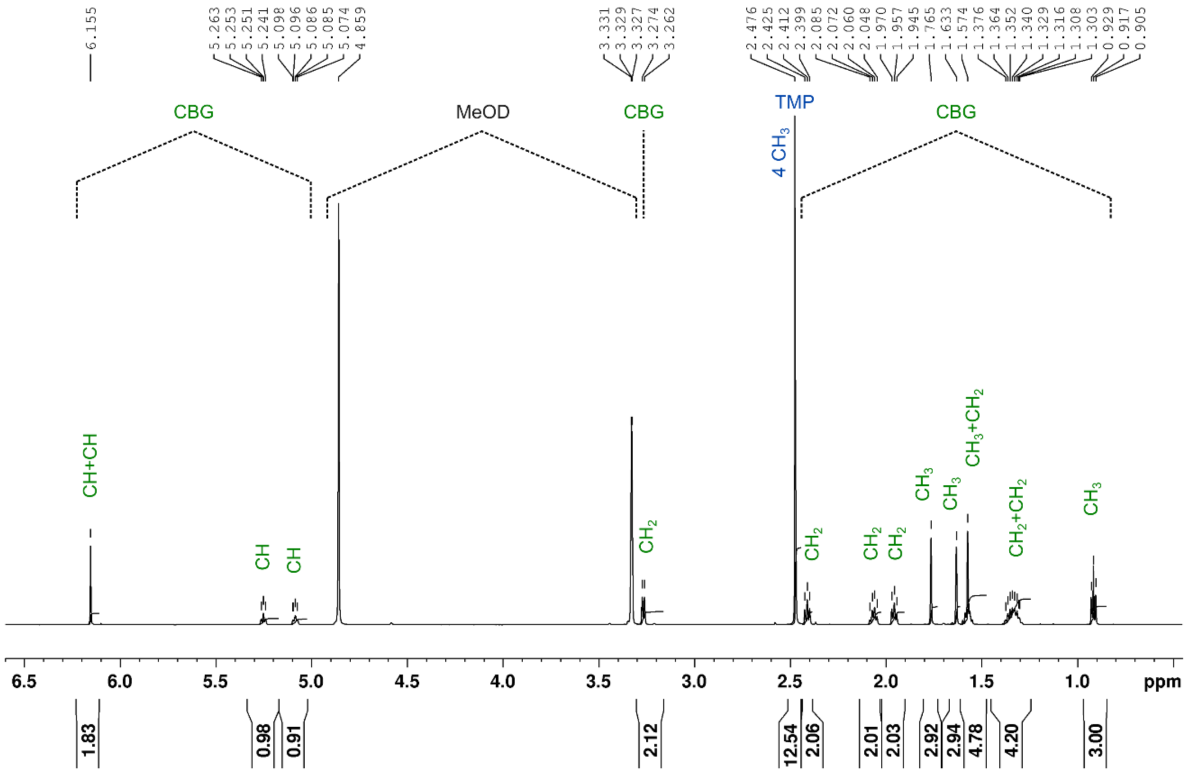

Figure S4 – ^{1}H NMR spectrum of a novel CBG–TMP solid phase, form I.

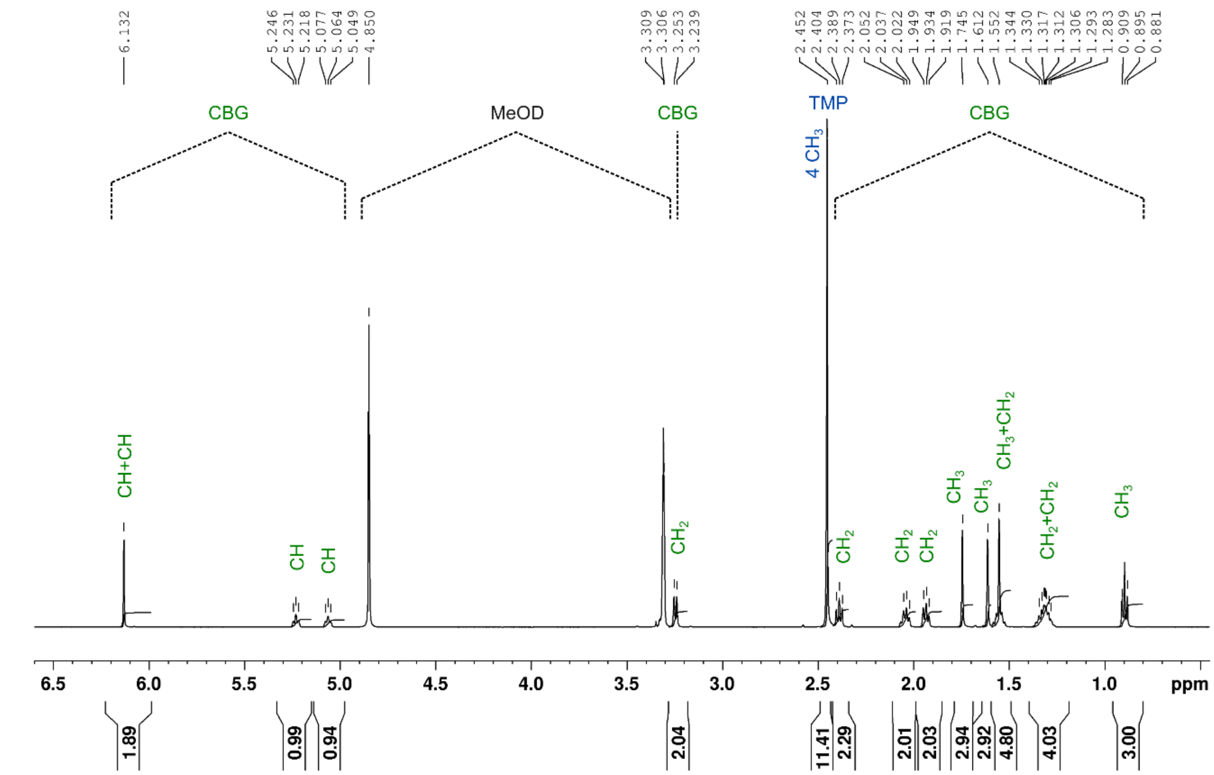

Figure S5 – ^{1}H NMR spectrum of a novel CBG–TMP solid phase, form II.

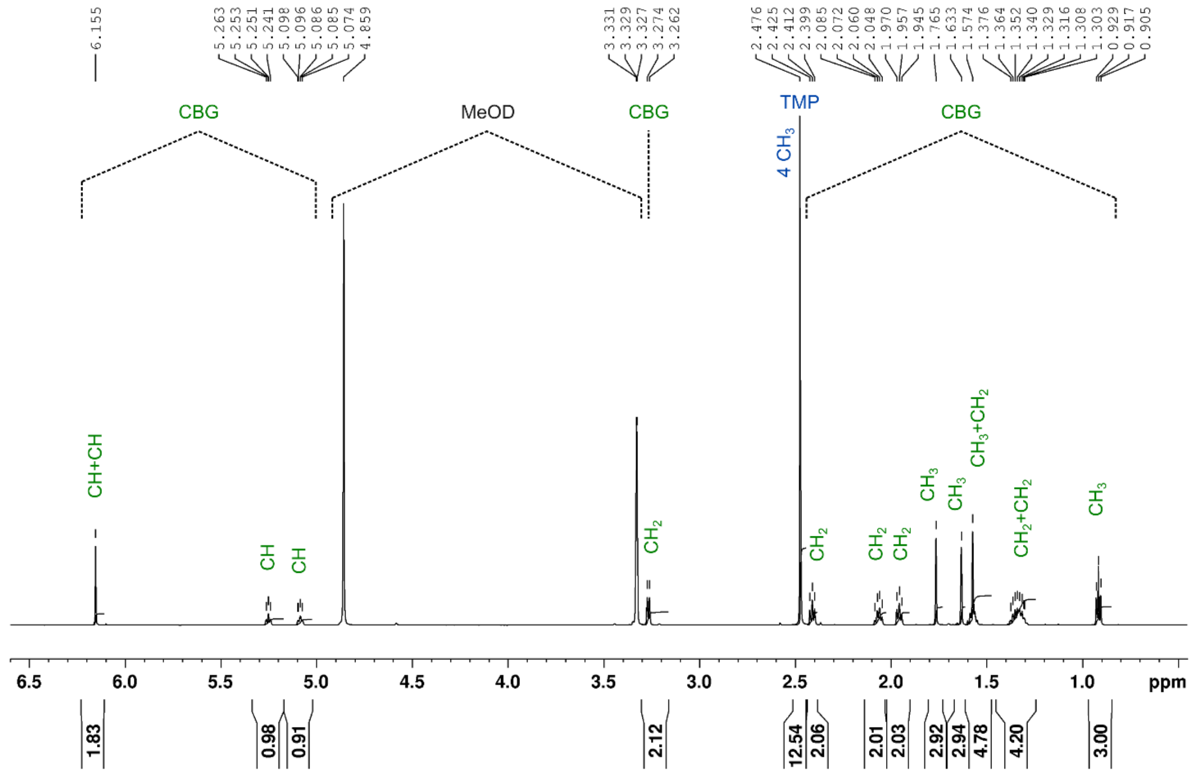

Figure S6 – ^{1}H NMR spectrum of a novel CBG–TMP solid phase, form III.

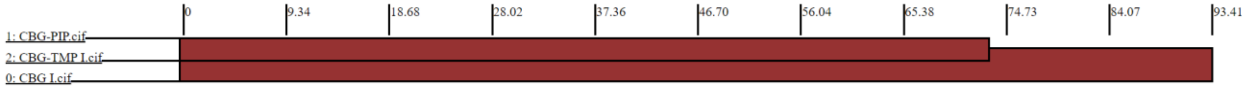

Figure S7 Similarity dendrogram – molecular packing of CBG

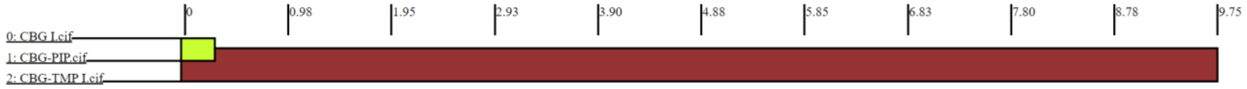

Figure S8 Similarity dendrogram – conformation of CBG

Table S6 Crystallography details

**Crystal data**	**CBG I**	**CBG - PIP**	**CBG – TMP I**
Chemical formula	$C_{21}H_{32}O_2$	$C_{25}H_{42}N_2O_2$	$C_{29}H_{44}N_2O_2$
M_r	316.48	402.62	452.68
Crystal system, space group	Orthorhombic, $P2_12_12_1$	Monoclinic, $P2_1/n$	Monoclinic, $P2_1/c$
Temperature (K)	95	95	95
a, *b*, *c* (Å)	4.5073 (1), 11.4901 (1), 36.7494 (3)	8.7047 (1), 26.7487 (1), 11.0627 (1)	8.8766 (1), 17.6450 (1), 17.2008 (1)
α, β, γ (°)	90, 90, 90	90, 111.2361 (8), 90	90, 91.5518 (6), 90
V (Å^3)	1903.23 (5)	2400.92 (4)	2693.13 (4)
Z	4	4	4
Radiation type	Cu *K*α	Cu *K*α	Cu *K*α
μ (mm^{-1})	0.53	0.54	0.53
Crystal size (mm)	0.79 × 0.22 × 0.10	0.53 × 0.44 × 0.21	0.52 × 0.46 × 0.27
Diffractometer	Oxford Diffraction SuperNova	Oxford Diffraction SuperNova	Oxford Diffraction SuperNova
Absorption correction	Multi-scan *CrysAlis PRO* (Rigaku Oxford Diffraction, 2017)	Multi-scan *CrysAlis PRO* (Rigaku Oxford Diffraction, 2017)	Multi-scan *CrysAlis PRO* (Rigaku Oxford Diffraction, 2017)
T_{min}, T_{max}	0.51, 0.95	0.18, 0.89	0.68, 0.86
No. of measured, indep. and observed [$I > 2.0\sigma(I)$] reflections	31358, 3871, 3798	84442, 4796, 4652	38765, 5365, 5138
R_{int}	0.043	0.067	0.026
$(\sin \theta/\lambda)_{max}$ (Å^{-1})	0.624	0.621	0.621

$R[F^2 > 2\sigma(F^2)]$, $wR(F^2)$, S	0.037, 0.096, 0.99	0.046, 0.128, 0.87	0.035, 0.091, 0.98
No. of reflections	3869	4796	5365
No. of parameters	218	316	307
No. of restraints	4	82	4
H-atom treatment	H atoms treated by a mixture of independent and constrained refinement	H atoms treated by a mixture of independent and constrained refinement	H atoms treated by a mixture of independent and constrained refinement
$\Delta\rho_{max}$, $\Delta\rho_{min}$ (e Å^{-3})	0.27, −0.20	0.52, −0.39	0.26, −0.19
Absolute structure	Parsons, Flack & Wagner (2013), 1496 Friedel Pairs	-	-
Absolute structure parameter	0.04 (7)	-	-
CCDC	2390063	2390061	2390062

Table S7 Selected geometric parameters (Å, °) of CBG I

O1—C2	1.3902 (19)	C10—C11	1.504 (3)
C2—C3	1.391 (2)	C10—C12	1.513 (3)
C2—C7	1.389 (2)	C12—C13	1.534 (3)
C3—C4	1.396 (2)	C13—C14	1.500 (3)
C4—C5	1.388 (2)	C14—C15	1.328 (3)
C4—C19	1.513 (2)	C15—C16	1.500 (3)
C5—C6	1.387 (2)	C15—C17	1.501 (3)
C6—C7	1.397 (2)	C19—C20	1.543 (2)
C6—O18	1.385 (2)	C20—C21	1.524 (2)
C7—C8	1.521 (2)	C21—C22	1.529 (3)
C8—C9	1.510 (2)	C22—C23	1.522 (3)
C9—C10	1.326 (3)		
O1—C2—C3	120.21 (15)	C8—C9—C10	128.54 (16)
O1—C2—C7	116.87 (15)	C9—C10—C11	124.55 (17)
C3—C2—C7	122.91 (15)	C9—C10—C12	119.95 (17)
C2—C3—C4	119.69 (15)	C11—C10—C12	115.50 (16)
C3—C4—C5	118.59 (15)	C10—C12—C13	114.12 (16)
C3—C4—C19	121.96 (15)	C12—C13—C14	111.90 (17)
C5—C4—C19	119.44 (15)	C13—C14—C15	129.0 (2)
C4—C5—C6	120.44 (16)	C14—C15—C16	124.1 (2)

C5—C6—C7	122.34 (15)	C14—C15—C17	121.2 (2)
C5—C6—O18	115.59 (14)	C16—C15—C17	114.64 (19)
C7—C6—O18	121.94 (15)	C4—C19—C20	112.97 (15)
C6—C7—C2	116.02 (15)	C19—C20—C21	113.53 (15)
C6—C7—C8	121.53 (15)	C20—C21—C22	113.41 (15)
C2—C7—C8	122.24 (15)	C21—C22—C23	112.88 (16)
C7—C8—C9	109.29 (14)		

Table S8 Hydrogen-bond geometry (Å, °) of CBG I

D—H···*A*	*D*—H	H···*A*	*D*···*A*	*D*—H···*A*
C9—H91···O18[i]	0.95	2.54	3.395 (3)	149
O1—H11···O1[ii]	0.814	1.887	2.700 (3)	177 (2)
O18—H181···O18[iii]	0.825	2.080	2.808 (3)	147 (2)

Symmetry codes: (i) *x*+1/2, −*y*+1/2, −*z*+1; (ii) *x*+1/2, −*y*+3/2, −*z*+1; (iii) *x*−1/2, −*y*+1/2, −*z*+1.

Table S9 Selected geometric parameters (Å, °) of CBG-PIP

O1—C2	1.3546 (15)	C14—C15	1.5023 (19)
C2—C3	1.4038 (17)	C15—C16	1.3334 (19)
C2—C8	1.3958 (19)	C16—C17	1.5060 (19)
C3—C4	1.4013 (17)	C16—C23	1.5078 (18)
C3—C14	1.5134 (18)	C17—C18	1.5359 (19)
C4—O5	1.3546 (15)	C18—C19	1.499 (2)
C4—C6	1.3959 (19)	C19—C20	1.328 (2)
C6—C7	1.3941 (18)	C20—C21	1.504 (2)
C7—C8	1.3940 (18)	C20—C22	1.495 (2)
C7—C9	1.516 (2)	N24—C25	1.4741 (16)
C9—C30	1.389 (3)	N24—C29	1.4754 (16)
C9—C31	1.692 (4)	C25—C26	1.5240 (17)
C30—C40	1.500 (3)	C26—N27	1.4723 (16)

C41—C31	1.519 (4)	N27—C28	1.4724 (16)
C41—C51	1.535 (4)	C28—C29	1.5222 (17)
C50—C60	1.506 (5)	C51—C61	1.494 (6)
C50—C40	1.512 (3)		
O1—C2—C3	116.48 (11)	C14—C15—C16	127.33 (12)
O1—C2—C8	122.79 (11)	C15—C16—C17	120.40 (12)
C3—C2—C8	120.73 (11)	C15—C16—C23	123.89 (13)
C2—C3—C4	118.21 (12)	C17—C16—C23	115.71 (12)
C2—C3—C14	120.78 (11)	C16—C17—C18	113.89 (12)
C4—C3—C14	120.97 (11)	C17—C18—C19	112.00 (12)
C3—C4—O5	116.95 (11)	C18—C19—C20	128.39 (14)
C3—C4—C6	120.92 (11)	C19—C20—C21	121.37 (14)
O5—C4—C6	122.10 (11)	C19—C20—C22	124.84 (13)
C4—C6—C7	120.37 (12)	C21—C20—C22	113.79 (13)
C6—C7—C8	119.19 (12)	C25—N24—C29	110.33 (9)
C6—C7—C9	120.48 (13)	N24—C25—C26	112.75 (10)
C8—C7—C9	120.29 (13)	C25—C26—N27	112.84 (10)
C2—C8—C7	120.53 (12)	C26—N27—C28	110.68 (10)
C7—C9—C30	125.0 (2)	N27—C28—C29	112.14 (10)
C7—C9—C31	103.76 (15)	C28—C29—N24	112.56 (10)
C9—C30—C40	112.77 (9)	C50—C40—C30	113.46 (9)
C31—C41—C51	113.61 (10)	C41—C31—C9	112.64 (9)
C60—C50—C40	114.28 (10)	C41—C51—C61	114.35 (10)
C3—C14—C15	111.82 (11)		

Table S10 Hydrogen-bond geometry (Å, °) of CBG-PIP

D—H···*A*	*D*—H	H···*A*	*D*···*A*	*D*—H···*A*
O1—H11···N24	0.879 (15)	1.816 (15)	2.680 (3)	167.0 (18)
C41—H412···C51^{i}	0.97	2.47	3.341 (3)	149
C31—H402···C51	1.06	1.73	2.556 (3)	131
O5—H51···C26^{ii}	0.867 (15)	2.542 (18)	3.152 (3)	128.1 (15)
O5—H51···N27^{ii}	0.867 (15)	1.842 (16)	2.679 (3)	161.7 (18)

Symmetry codes: (i) −*x*, −*y*+2, −*z*; (ii) *x*−3/2, −*y*+3/2, *z*−1/2.

Table S11 Selected geometric parameters (Å, °) of CBG-TMP I

O1—C2	1.3665 (11)	C14—C15	1.5020 (14)
C2—C3	1.3933 (14)	C14—C16	1.5022 (14)
C2—C7	1.3965 (13)	C19—C20	1.5347 (14)
C3—C4	1.3946 (13)	C20—C21	1.5267 (14)
C4—C5	1.3930 (13)	C21—C22	1.5213 (15)
C4—C19	1.5092 (13)	C22—C23	1.5194 (16)
C5—C6	1.3946 (13)	N24—C25	1.3418 (13)
C6—C7	1.3990 (13)	N24—C29	1.3374 (13)
C6—O18	1.3627 (11)	C25—C26	1.3967 (13)
C7—C8	1.5111 (13)	C25—C33	1.5009 (14)
C8—C9	1.5091 (13)	C26—N27	1.3416 (13)
C9—C10	1.3327 (14)	C26—C32	1.5013 (14)
C10—C11	1.5125 (13)	N27—C28	1.3400 (13)
C10—C17	1.5056 (13)	C28—C29	1.4010 (13)
C11—C12	1.5435 (14)	C28—C31	1.5004 (13)
C12—C13	1.5031 (15)	C29—C30	1.5004 (13)
C13—C14	1.3325 (14)		
O1—C2—C3	121.61 (8)	C13—C14—C15	121.53 (10)
O1—C2—C7	116.79 (8)	C13—C14—C16	123.72 (10)
C3—C2—C7	121.59 (8)	C15—C14—C16	114.73 (9)
C2—C3—C4	120.06 (9)	C4—C19—C20	112.31 (8)
C3—C4—C5	119.18 (9)	C19—C20—C21	113.79 (8)
C3—C4—C19	120.62 (9)	C20—C21—C22	113.12 (9)
C5—C4—C19	120.17 (8)	C21—C22—C23	112.57 (10)
C4—C5—C6	120.23 (8)	C25—N24—C29	118.73 (8)
C5—C6—C7	121.34 (9)	N24—C25—C26	120.76 (9)
C5—C6—O18	122.26 (8)	N24—C25—C33	117.56 (9)
C7—C6—O18	116.40 (8)	C26—C25—C33	121.67 (9)
C6—C7—C2	117.60 (8)	C25—C26—N27	120.52 (9)
C6—C7—C8	120.98 (8)	C25—C26—C32	121.61 (9)
C2—C7—C8	121.42 (8)	N27—C26—C32	117.87 (9)
C7—C8—C9	111.59 (8)	C26—N27—C28	118.78 (8)

C8—C9—C10	127.67 (9)	N27—C28—C29	120.60 (9)
C9—C10—C11	120.54 (9)	N27—C28—C31	117.89 (9)
C9—C10—C17	123.68 (9)	C29—C28—C31	121.50 (9)
C11—C10—C17	115.75 (8)	C28—C29—N24	120.61 (9)
C10—C11—C12	114.87 (8)	C28—C29—C30	121.64 (9)
C11—C12—C13	112.09 (8)	N24—C29—C30	117.72 (8)
C12—C13—C14	126.16 (10)		

Table S12 Hydrogen-bond geometry (Å, °) of CBG-TMP I

D—H···*A*	*D*—H	H···*A*	*D*···*A*	*D*—H···*A*
O18—H181···N24	0.863 (12)	1.984 (13)	2.8244 (14)	164.2 (14)
O1—H11···N27^{i}	0.850 (12)	2.010 (13)	2.8150 (14)	157.7 (13)
Symmetry code: (i) x−1, −y+3/2, z+1/2.				

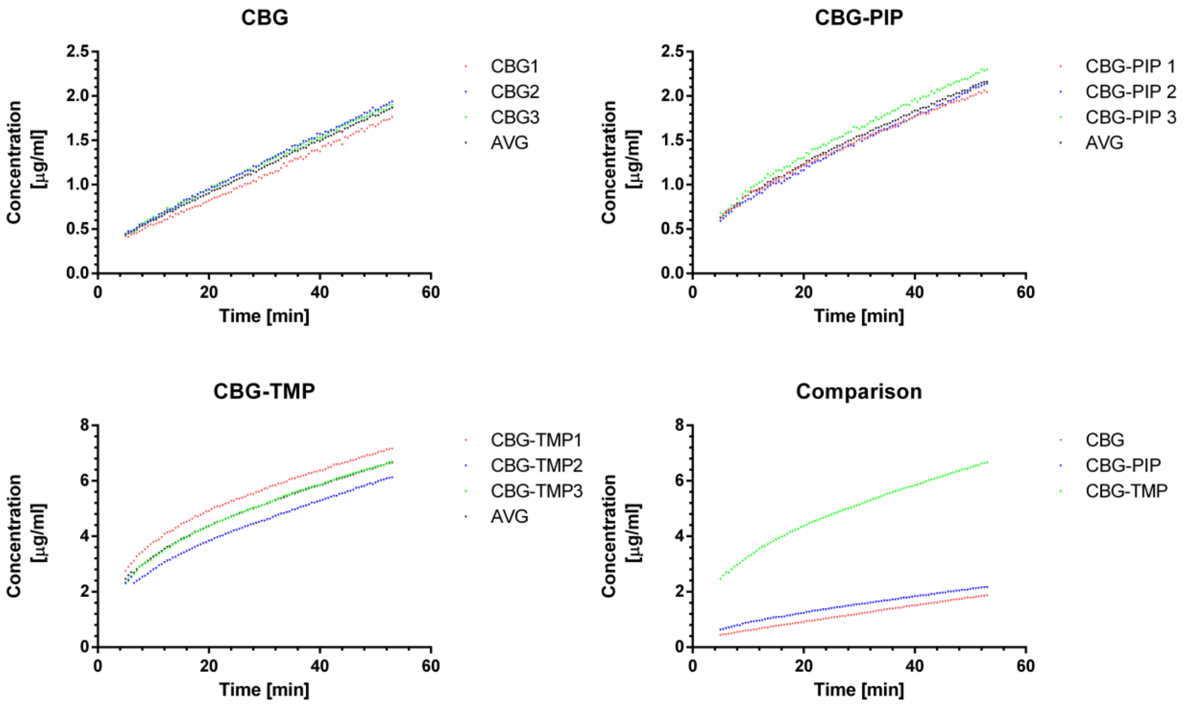

Figure S9 IDR curves

Table S13 Lattice energy calculation results

Lattice energy	CBG I	CBG - PIP	CBG - TMP I
Total	-209.613	-107.865	-134.64
Electrostatic	-20.187	-8.758	-8.923
van der Waals	-146.143	-75.723	-103.088
H-bonding	-43.282	-23.384	-22.629

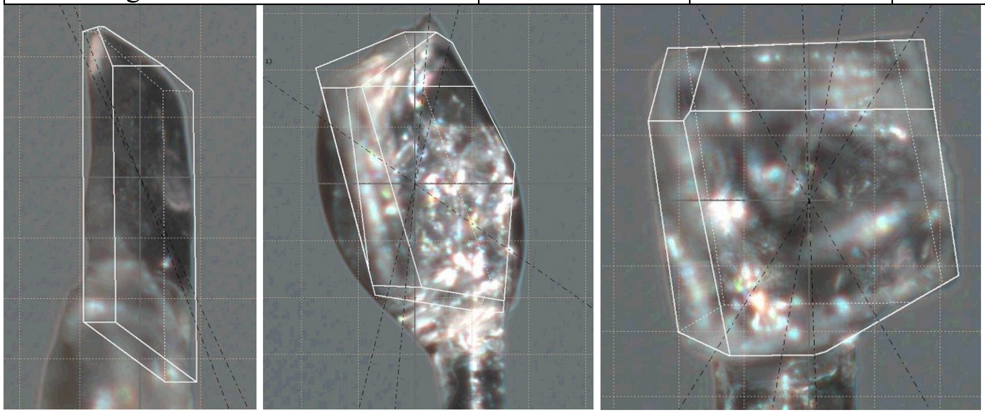

Fig. S10 Single-crystals used for the diffraction experiments. Left – CBG I, middle – CBG-PIP, right – CBG-TMP I

Table S14 Smallest (S), medium (M) and largest (L) dimensions of the calculated and experimental crystals. Units: measured – mm, calculated - arbitrary

	CBG I		CBG - PIP		CBG - TMP I	
	measured	calculated	measured	calculated	measured	calculated
S	0.079	2	0.213	6.9	0.274	6.1
M	0.213	6.1	0.438	7.1	0.457	6.1
L	0.64	12.3	0.532	10.1	0.522	7.8
S / M	0.370892	0.32786885	0.48630137	0.97183099	0.599562	1
M / L	0.332813	0.49593496	0.823308271	0.7029703	0.875479	0.782051

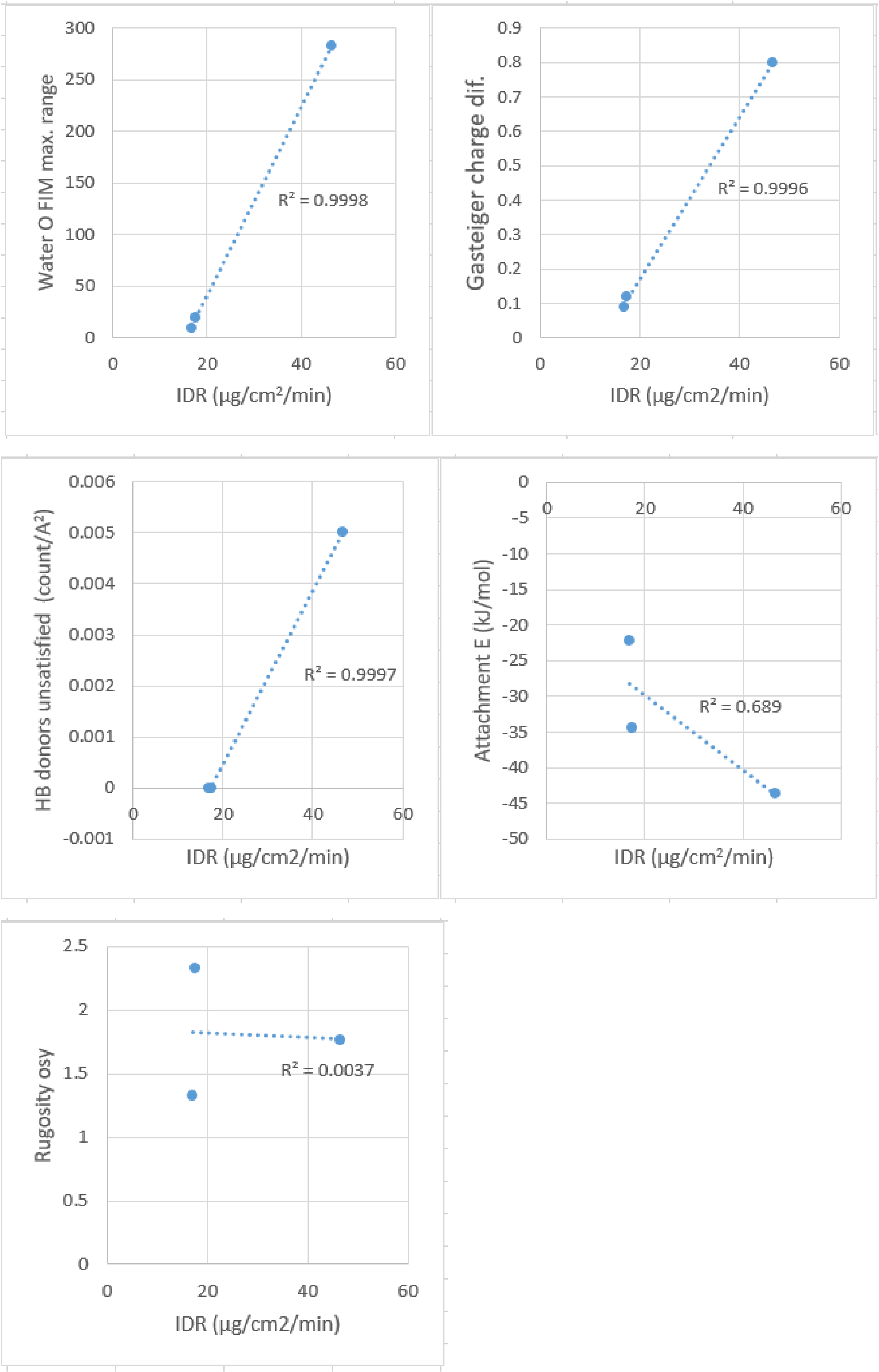

Figure S11 Correlations between the intrinsic dissolution rates (IDR) of CBG and its cocrystals with their crystal surface properties
